# The splicing factor RBM17 drives leukemic stem cell maintenance by evading nonsense-mediated decay of pro-leukemic factors

**DOI:** 10.1038/s41467-022-31155-0

**Published:** 2022-07-04

**Authors:** Lina Liu, Ana Vujovic, Nandan P. Deshpande, Shashank Sathe, Govardhan Anande, He Tian Tony Chen, Joshua Xu, Mark D. Minden, Gene W. Yeo, Ashwin Unnikrishnan, Kristin J. Hope, Yu Lu

**Affiliations:** 1 Department of Medicine, Faculty of Health Sciences, McMaster University, Hamilton, ON Canada; 2 Department of Biochemistry and Biomedical Sciences, Faculty of Health Sciences, McMaster University, Hamilton, ON Canada; 3 Department of Medical Biophysics, University of Toronto, Toronto, ON Canada; 4 Princess Margaret Cancer Centre, University Health Network, Toronto, ON Canada; 5 Adult Cancer Program, Lowy Cancer Research Centre, University of New South Wales, Sydney, NSW Australia; 6 Prince of Wales Clinical School, University of New South Wales, Sydney, NSW Australia; 7 Department of Cellular and Molecular Medicine, Stem Cell Program and Institute for Genomic Medicine, University of California at San Diego, San Diego, CA USA

**Keywords:** RNA-binding proteins, Cancer stem cells, Acute myeloid leukaemia

## Abstract

Chemo-resistance in acute myeloid leukemia (AML) patients is driven by leukemic stem cells (LSCs) resulting in high rates of relapse and low overall survival. Here, we demonstrate that upregulation of the splicing factor, RBM17 preferentially marks and sustains LSCs and directly correlates with shorten patient survival. *RBM17* knockdown in primary AML cells leads to myeloid differentiation and impaired colony formation and in vivo engraftment. Integrative multi-omics analyses show that *RBM17* repression leads to inclusion of poison exons and production of nonsense-mediated decay (NMD)-sensitive transcripts for pro-leukemic factors and the translation initiation factor, EIF4A2. We show that EIF4A2 is enriched in LSCs and its inhibition impairs primary AML progenitor activity. Proteomic analysis of *EIF4A2*-depleted AML cells shows recapitulation of the *RBM17* knockdown biological effects, including pronounced suppression of proteins involved in ribosome biogenesis. Overall, these results provide a rationale to target RBM17 and/or its downstream NMD-sensitive splicing substrates for AML treatment.

## Abstract (web summary)

Leukemic stem cells (LSCs) drive chemoresistance and relapse in acute myeloid leukemia. Here, the authors show that the splicing factor RBM17 supports LSCs through avoiding nonsense-mediated decay of pro-leukaemic factors such as the translation initiation factor EIF4A2.

## Introduction

Acute myeloid leukemia (AML) is a malignant hematopoietic disorder with dysregulated clonal expansion of mutant undifferentiated myeloid progenitor cells, and accounts for approximately 30% of adult leukemias^1^. Despite significant advances in cancer therapeutics in recent years, adult AML patients continue to display chemo-resistance at presentation, high relapse rates, and a 5-year overall survival rate less than 25%^1^. AML is maintained by relatively rare populations of leukemic stem cells (LSCs) that are responsible for seeding and propagating the disease^2–4^ and possess stem cell-like characteristics including the capacity for self-renewal, differentiation potential (albeit limited), and relative quiescence^5–7^. This latter property of LSCs, as well as their possession of natural resistance mechanisms such as drug efflux pumps, contributes to their intrinsic resistance to conventional chemotherapies that target proliferating cells. In addition, multiple studies have demonstrated that patients whose bulk AML cells have an elevated LSC gene expression signature have worse clinical outcomes^5,8^, suggesting that heightened LSC activity correlates with poor efficacy of conventional therapy.

LSCs and other primitive leukemic cells are generally thought to be transformed from hematopoietic stem cells (HSCs) or committed progenitor cells and very often share the same surface markers (CD34^+^CD38^-^ and CD34^+^, respectively) and similar mechanisms that support the self-renewal^9^ of their primitive normal counterparts. These similarities make it difficult to specifically target primitive leukemic cells for drug development. Recently a 78-patient study defined a panel of 17 LSC signature genes, whose expression levels were shown to be predictive of response to treatment and overall survival for patients treated with daunorubicin and cytarabine^8^. Despite these findings, there has been limited success in the effort to specifically target primitive leukemic cells for AML treatment. Therefore, it is essential to gain a more comprehensive understanding of the mechanistic elements that underpin primitive leukemic cell function and that, as such, may represent important and novel therapeutic targets in AML.

Alternative splicing (AS) is one of the major contributors to proteome diversity and is thus tightly controlled throughout normal development^10^. AS is a complex process that involves a variety of regulatory *trans-acting* splicing factors and responsive *cis*-acting RNA elements, which act together to determine splice site selection and alternative exon usage^11,12^. Aberrant alternative splicing is recognized as a key driver of cancer, with many of the hallmark processes of cancer being regulated by tumor-specific splice variants^13^. Dysregulated AS can either alter transcript stability, resulting in changes in protein levels or affect coding potential, leading to expression of proteins with distinctly different functions. In the context of AML, a genome-wide analysis of aberrant AS patterns showed that approximately one third of genes are differentially spliced in the primitive CD34^+^ cells of AML patients compared to those obtained from normal controls, suggesting that such genes are involved in processes key to cellular function^14^. LSCs also have a unique AS profile when compared to normal aging HSCs, including a switch to pro-survival isoforms, which enhances their maintenance^15^. For example, mis-splicing of GSK3β enhances the malignant transformation from human pre-leukemic progenitors into self-renewing LSCs^16^. Aberrations in AS can result from somatic mutations in splicing factors or in *cis-acting* motifs within exons or introns, or abnormal expression of splicing factors. Analyses of the genomic landscape of AML patients have discovered recurrent mutations in splicing factors SRSF2, SF3B1 and U2AF1, however these mutations are only found in approximately 10% of AML patients studied^17–19^. Given that abnormal AS is also prevalent in AML patients with no obvious mutations in RNA splicing genes^14^, it is critical to study the deregulation of splicing factor expression and their underlying mechanism in AML and primitive LSC. In the present study, we focused on aberrant AS in human primitive AML biology and show that RBM17 is preferentially expressed in progenitors and LSCs and enacts within these cells an AS program that is critical for supporting their maintenance.

## Results

### RBM17 expression is associated with primitive AML cells and adverse AML prognosis

**Fig. 1:**
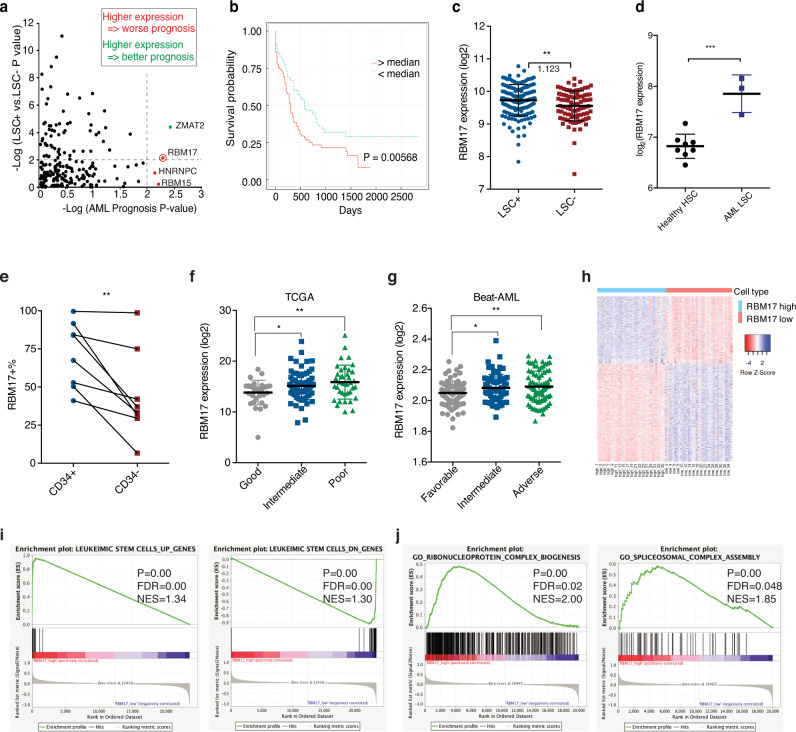
Heightened expression of RBM17 correlates with poor prognosis in human AML patients. **a** –log10 p-value (two-tailed Student’s *t* test) of each mRNA splicing factor expression in LSC-enriched vs LSC-depleted subsets from 78 AML patients (*y* axis) and their correlation (-log10 *p*-value) (log-rank test) with AML patients’ overall survival (*x* axis). **b** Kaplan–Meier curves showing outcomes of AML patients from the TCGA with above (*n* = 124) vs below (*n* = 155) median expression of RBM17. (*P* = 0.00568, log-rank test). **c** *RBM17* transcript level in LSC-enriched (*n* = 138) vs LSC-depleted (*n* = 89) subsets from 78 AML patient samples (GSE76008). Average fold change of *RBM17* expression in LSC-enriched over LSC-depleted subsets is indicated in the figure. Data are presented as mean ± SD, two-tailed Student’s *t* test. **d** Gene expression data (GSE35008) from sorted AML bone marrow samples were compared with data from healthy controls and revealed significantly increased *RBM17* expression in AML LT-HSCs (Lin^–^CD34 + CD38^–^CD90 + , AML with normal karyotype, *n* = 3) compared with healthy control (*n* = 4). Data shown as mean ± SD, two-tailed Student’s *t* test. **e** Intracellular flow cytometric measurements of RBM17 protein level in the primitive CD34^+^ subset vs the committed CD34^-^ subsets from 8 primary AML samples. *P* = 0.0092, *P* value was calculated using paired t-test, two-tailed. **f** Expression of *RBM17* in 152 AML specimens and each molecular genetic risk group from the TCGA-LAML cohort (Good: *n* = 38; Intermediate: *n* = 76; Poor: *n* = 38). Data are presented as mean ± SD, two-tailed Student’s *t* test, *P*(Good vs Intermediate) = 0.0196, *P*(Good vs Poor)=0.0051. **g** Expression of *RBM17* in 236 AML specimens and each molecular genetic risk group from Beat AML cohort (Favorable: *n* = 85, Intermediate: *n* = 68, Adverse: *n* = 83). Data are presented as mean ± SD, two-tailed Student’s *t* test, *P*(Favorable vs Intermediate) = 0.0188, *P*(Favorable vs Adverse)=0.0041. **h** A heatmap showing the expression of differentially expressed transcripts identified from *RBM17*-high AML cases versus *RBM17*-low AML cases. **i**, **j** Gene set enrichment analysis (GESA) of the gene signature of high-*RBM17* AML cases compared with previously published **i** LSC signatures and **j** ribonucleoprotein complex biogenesis and spliceosomal complex assembly pathways. The significance of NES was calculated using Kolmogorov–Smirnov statistics. *P < 0.05, **P < 0.01, ***P < 0.001. Source data are provided as a Source Data file.

Previous studies examining the link between aberrant splicing and AML have focused on spliceosome genes with somatic mutations in AML patients or with abnormal expression levels in bulk AML samples^20,21^. To more broadly profile splicing factors that may mediate aberrant alternative splicing independently of mutations in AML cells, we performed a data-mining survey of 203 known mRNA splicing factors (members of the “mRNA splicing” and “mRNA alternative splicing” Gene Ontology (GO) categories^22^ (Supplementary Data 1). Strikingly, RNA-binding motif protein 17 (RBM17) was the only splicing factor that was both significantly elevated (*P* = 0.0086) in the LSC-enriched (LSC + ) vs LSC-devoid (LSC-) subsets from 78 karyotypically normal AML patient samples (GSE76008)^8^ and strongly linked (*P* = 0.00568) to poor AML prognosis^19^ (Fig. 1a–c). To validate the link between *RBM17* expression and AML prognosis, we analyzed two additional independent cohorts of AML patients. Above median levels of *RBM17* expression is significantly linked to poor outcome of AML patients from the Leucegene dataset (*P* = 0.034)^23^ and showed a negative prognostic trend in BeatAML datasets (*P* = 0.059)^17^ (Supplementary Fig. 1a-b). Next, we analyzed the published gene expression profiles of purified LT-HSCs (Lin^-^CD34^+^CD38^-^CD90^+^) from healthy donors and AML samples with normal karyotype (GSE35008)^24^, and observed that RBM17 is expressed at significantly higher levels in AML LSCs compared to normal LT-HSCs (Fig. 1d). We went on to validate these results in 8 primary AML samples, along with a unique OCI-AML-8227 AML cell line, which was derived from a primary AML sample and retains an LSC-driven hierarchy^25^. We found that the *RBM17* transcript level is significantly upregulated in the primitive cell subset (CD34^+^) as compared to the committed cell subset (CD34^-^) in OCI-AML-8227 cells (Supplementary Fig. 1c). In keeping with the increased level of mRNA, RBM17 protein is 1.68 fold higher in the LSC-enriched primitive cell subsets (CD34^+^) of primary AML patient samples (Supplementary Table 1) compared to the more committed cell subsets (CD34^-^) (Fig. 1e, Supplementary Fig. 1d-e), providing further support that elevated RBM17 preferentially marks the primitive compartments of human AML.

To further characterize RBM17 in AML patient cells, we analyzed its expression in the TCGA-LAML (https://portal.gdc.cancer.gov/projects/TCGA-LAML) and BeatAML datasets and found that *RBM17* expression was significantly higher in both poor/adverse and intermediate molecular genetic risk groups compared with the good/favorable molecular genetic risk group (Fig. 1f, g). Next, to investigate the gene expression signature of AML patients with high expression of *RBM17*, we ranked AML patient samples from the GSE76008 dataset based on *RBM17* expression level and defined the top 15% (35 of 221) as *RBM17*-high cases, and the bottom 15% (35 of 221) as *RBM17*-low cases. In total, we identified 832 differentially expressed genes (false discovery rate (FDR) ≤ 0.05, flod change (FC) ≥ 2 or ≤0.5), including 336 transcripts more abundant, and 496 transcripts less abundant in AML patient samples with higher *RBM17* expression (Fig. 1h). Interestingly, of these genes, 82.1% of those co-regulated with *RBM17* are more highly expressed in LSCs and 73.2% of the genes anti-correlated with RBM17 are expressed at lower levels in LSCs, indicating that expression of *RBM17* and its associated co-regulated genes is highly correlated with the LSC gene expression signature (*R*^2^ = 0.7584, *P* < 0.0001) (Supplementary Fig. 1f). To confirm this hypothesis, we carried out Gene Set Enrichment Analysis (GSEA) with the LSC gene set that contains upregulated (UP) and downregulated (DN) genes in LSCs^8^, and showed highly significant enrichment of the LSC signature gene set in genes co-regulated with RBM17 in AML patients (Fig. 1i). In addition, GSEA also revealed significant enrichment of genes involved in ribonucleoprotein complex biogenesis and spliceosomal complex assembly (FDR ≤ 0.05) (Fig. 1j, Supplementary Table 2) in the set of genes co-regulated with *RBM17*. These results together suggest that RBM17 could have an important role in supporting primitive leukemic cell functions.

### RBM17 knockdown impairs the stem and progenitor colony-forming potential of primary AML

**Fig. 2:**
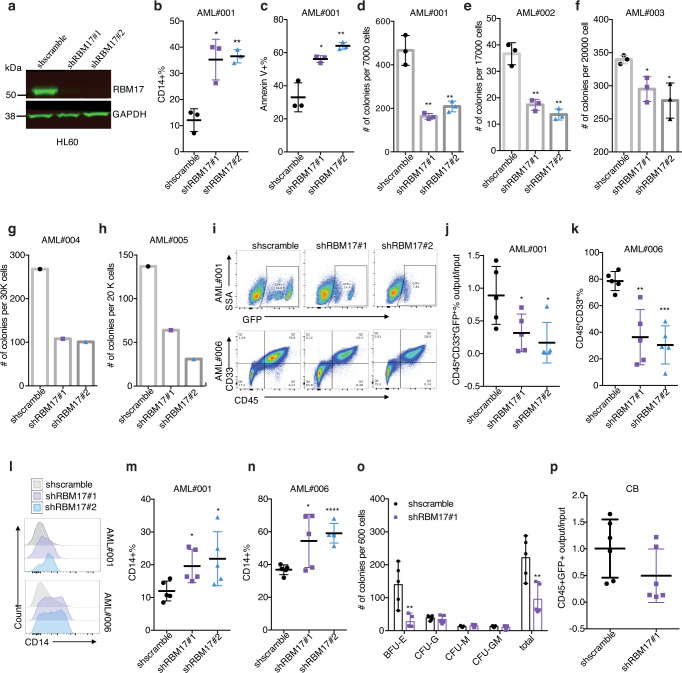
RBM17 is required to support primitive leukemic cell functions. **a** Western blot validation of *RBM17* knockdown (KD) in transduced GFP^+^ HL60 cells. *n* = 3 independent experiments. **b**, **c** Flow cytometric evaluation of myeloid differentiation (P(shscramble vs shRBM17#1)=0.0108, P(shscramble vs shRBM17#2)=0.0011)) (**b**) and apoptosis (P(shscramble vs shRBM17#1)=0.0111, P(shscramble vs shRBM17#2)=0.0039) (**c**) following *RBM17* knockdown in primary AML cells. *n* = 3, mean ± SD, two-tailed Student’s *t* test. **d**–**h** Colony formation capacity assessed in 5 AML samples following *RBM17* KD. *n* = 3, mean ± SD, two-tailed Student’s *t* test. **i** Representative flow cytometry plots showing the GFP^+^ cell percentage in the human cell population post-transplant for AML#001 (top) and total human grafts (CD45^+^CD33^+^ cell percentage) post-transplant for AML#006 (bottom). **j** Quantification of AML engraftment after 9 weeks (#001) in whole bone marrow. Shown is the ratio of the GFP^+^ cell percentage in the human cell population post-transplant to the initial pre-transplant GFP^+^ cell percentage. Data are presented as mean ± SD, *n* = 5, two-tailed Student’s *t* test, P(shscramble vs shRBM17#1)=0.0415, P(shscramble vs shRBM17#2)=0.0173. **k** AML engraftment (#006) after 12 weeks in bone marrow. Shown is the percentage of human (CD45^+^) myeloid cells (CD33^+^) found in bone marrow. *n* = 5, mean ± SD, two-tailed Student’s *t* test, P(shscramble vs shRBM17#1)=0.0026, P(shscramble vs shRBM17#2)=0.0002. **l**–**n** Representative histogram (**l**) and quantification (**m**, **n**) of flow cytometric immunophenotyping of myeloid differentiation in post-transplant grafts from **j**, **k**. *n* = 5, mean ± SD, two-tailed Student’s *t* test. **o** CFU output from transduced Lin^-^ CD34^+^ cord blood (CB). *n* = 5, mean ± SD, two-tailed Student’s *t* test. **p** Relative engraftment of CD34^+^CD38^-^ enriched normal CB HSC with and without *RBM17* knockdown. Shown is the ratio of the GFP^+^ cell percentage in the human cell population post-transplant to the initial pre-transplant GFP^+^ cell percentage. *n* = 6, mean ± SD, two-tailed Student’s *t* test. *P < 0.05, **P < 0.01, ***P < 0.001, ****P < 0.0001. Source data are provided as a Source Data file.

To examine the functional roles of RBM17 in primitive AML cells, we knocked down *RBM17* using short hairpin RNA (shRNA) in human AML cell lines and patient samples. Briefly, we designed lentiviruses encoding GFP (transduction marker) along with two independent shRNAs, both of which resulted in efficient knockdown of RBM17 after transduction in multiple AML cell lines (shRNA #1 and #2) (Fig. 2a, Supplementary Fig. 2a). Interestingly, RBM17 knockdown inhibited AML cell growth (Supplementary Fig. 2b–d) and induced myeloid differentiation in the HL60 AML cell line (Supplementary Fig. 2e), the aforementioned OCI-AML-8227 cell line (Supplementary Fig. 2f), and primary AML cells (Fig. 2b, Supplementary Fig. 2g–i). In addition, RBM17 knockdown in primary AML cells significantly impaired survival (Fig. 2c, Supplementary Fig. 2j–l) and reduced colony numbers (Fig. 2d–h, Supplementary Fig. 2m), indicating that RBM17 is required for supporting AML survival and progenitor colony-forming potential in vitro. Next, to directly assess the role of RBM17 in LSC growth and survival in vivo, we performed xenograft studies using shRBM17-transduced primary AML specimens. We conducted output/input analysis of the GFP positivity of human (CD45^+^) cells for AML sample #001 (Fig. 2i, j, Supplementary Fig. 2n) where we achieved a ~35% infection rate, and analysis of the percentage of total CD45^+^ cells for AML sample #006 where transduction reached saturation (>85%) (Fig. 2i, k, Supplementary Fig. 2o). We observed that RBM17 knockdown in these two primary AML samples greatly impeded AML engraftment in transplanted immunodeficient mice (Fig. 2i–k, Supplementary Fig. 2p). Analysis of the resulting grafts revealed that RBM17 knockdown induced myeloid differentiation in vivo as shown by an increased percentage of mature CD14^+^ cells in shRBM17^+^ grafts compared to controls (Fig. 2l–n, Supplementary Fig. 2q) and decreased the output number of CD45^+^GFP^+^CD34^+^ cells (Supplementary Fig. 2r). Together, these findings indicate *RBM17* depletion disrupts primitive AML cell function through enhancing differentiation and inhibiting colony-forming and engraftment capacities.

### RBM17 knockdown impairs erythropoiesis without significantly affecting the engraftment potential of human HSPCs

Intriguingly, in contrast to the situation for the malignant hierarchy, the level of RBM17 in normal HSCs is lower than that in more committed cell populations in the normal hematopoietic system^26^ (Supplementary Fig. 2s). To investigate the role of RBM17 in normal hematopoietic stem and progenitor cells (HSPCs) in vitro, we knocked down *RBM17* in lineage depleted (Lin^-^) cord blood (CB) cells and performed colony forming unit (CFU) assays. Although the number of burst-forming unit-erythroid colonies (BFU-E) was significantly reduced upon RBM17 repression, the number of granulocyte colonies (CFU-G), megakaryocyte colonies (CFU-M), and granulocyte/macrophage colonies (CFU-GM) were not impeded (Fig. 2o), suggesting that RBM17 loss is more indispensable for erythropoiesis but has limited adverse impact on myeloid progenitor cells in vitro. Consistent with this observation, analysis of RBM17 expression during normal hematopoiesis shows that the expression of RBM17 is highest in the erythroid lineage (Supplementary Fig. 2s). Next, to assess the effect of RBM17 knockdown on the engraftment ability of HSCs, we depleted RBM17 in CB Lin^-^CD34^+^CD38^-^ cells and conducted in vivo xenotransplantation assays. We demonstrated that *RBM17* knockdown did not cause a significant loss of HSC-derived long-term engraftment capacity compared to control (Fig. 2p) nor did it impair the output number of normal primitive HSPCs (CD45^+^GFP^+^CD34^+^) in vivo (Supplementary Fig. 2t). These results together suggest that depletion of RBM17 impairs the colony forming ability and engraftment capacity of AML, but relatively spares normal HSPCs.

### RBM17 controls alternative splicing of genes involved in multiple pathways in AML cells

**Fig. 3:**
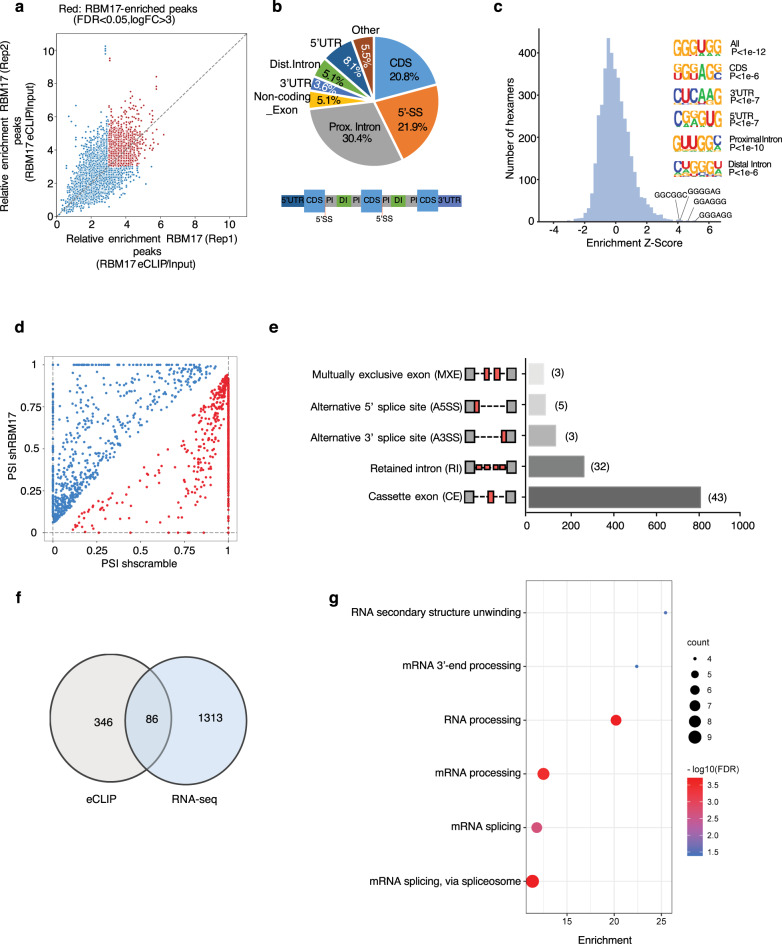
Identification of RNA targets of and alternative splicing variants enforced by RBM17 in AML. **a** eCLIP-seq analysis of RBM17 binding sites in K562 cells. Input-normalized peak signals are shown as log2 fold change. Red points indicate eCLIP-enriched RBM17 peaks (FDR < 0.05 and log2 (FC) > 3) in biological replicates. **b** Significantly enriched reproducible binding sites or RBM17 across the transcriptome. **c** Hexamer enrichment for RBM17 binding peaks in K562 cells based on eCLIP-seq. The top 4 enriched motifs are shown on the x axis. Insert shows enriched motifs for different genomic regions. **d** Scatterplot of splicing events promoted (blue circles) and repressed (red circles) by *RBM17* knockdown (shRBM17) in K562 cells compared to control shscramble with a cutoff FDR < 0.1 and ΔPSI > 0.05. Splicing change is quantified using ΔPSI (percent spliced in). **e** Quantification of types of AS events aff**e**cted by RBM17, as revealed by analysis of RNA-seq data. AS events are labeled as cassette exon (CE), retained intron (RI), alternative 3’ splice site (A3SS), alternative 5’ splice site (A5SS) and mutually exclusive exon (MXE). Number of splicing events directly bound by RBM17 is indicated beside the bar of each splicing type. **f** Overlay of transcripts between RBM17 eCLIP-seq analysis and RNA-seq splicing analysis. **g** GO enrichment analysis of terms enriched in splicing events directly bound by RBM17.

To understand the molecular mechanisms that underlie the supporting role of the splicing factor RBM17 in AML, we first performed RBM17 enhanced crosslinking immunoprecipitation (eCLIP)-seq in the K562 myeloid leukemia cell line to identify genome-wide RNA targets bound by RBM17 (Supplementary Fig. 3a). eCLIP-seq analysis identified 866 significantly enriched reproducible binding peaks for RBM17 in the genome using a cutoff of FDR < 0.05 and log2 (FC) > 3 (over size-matched input control), which corresponded to 432 annotated transcripts (Fig. 3a and Supplementary Data 2). Of these transcripts 93.1% are protein coding genes (Supplementary Fig. 3b). The majority of these peaks are within the coding sequence (CDS, 20.8%), 5’-splice site (5’-SS, 21.9%), or proximal intronic regions which are closer to intron/exon boundaries (30.4%) (Fig. 3b). The enrichment of RBM17 binding peaks around splice sites is consistent with its known function as a splicing regulator. Through motif analysis, we also identified that highly G-enriched motifs mapped to RBM17-binding sites (Fig. 3c). GO analysis of enriched binding sites further showed that transcripts involved in mRNA splicing, RNA processing, translation initiation, DNA repair and protein ubiquitination are preferentially bound by RBM17 (Supplementary Fig. 3c).

To further identify possible functional consequences of these interactions, we analyzed a published ENCODE RNA-seq dataset of shRBM17 (GSE88633) vs Control (GSE88047) transduced K562 cells^27^. We discovered AS events that are affected by *RBM17* knockdown in K562 cells (FDR < 0.1, Δpercent spliced in (PSI) > 0.05) (Supplementary Data 3), among which exon inclusions of 705 splicing events were supported by RBM17 while the other 633 splicing events were repressed by RBM17 (Fig. 3d). We also observed that RBM17 can be involved in many types of AS events, including Cassette exon (CE), Retained intron (RI), Alternative 3’ splice site (A3SS), Alternative 5’ splice site (A5SS) and Mutually exclusive exon (MXE), with cassette exons being the most affected (Fig. 3e). We next validated that multiple RBM17-regulated AS events are shared by K562 and HL60 cell lines (Supplementary Fig. 3d–f). GO analysis revealed that RBM17-affected AS events are strongly enriched in pathways related to gene transcription, DNA repair, cell division and RNA processing^28^ (Supplementary Fig. 3g). Next, by overlapping the eCLIP-seq and RNA-seq data, we identified 86 splicing events that are both bound and controlled by RBM17 (Fig. 3e, f, Supplementary Data 4), suggesting that RBM17 can regulate splicing through direct and specific association with the pre-mRNA of these transcripts. Such relationships are in contrast to the previously reported role of RBM17 as a spliceosome component with no preference for its RNA substrate sequences^29^. Further GO analysis showed that these RBM17 directly regulated AS events are significantly enriched in processes related to RNA-processing, RNA splicing, and RNA secondary structure unwinding (Fig. 3g). These results together suggest that RBM17 regulates a network of RNA-processing proteins involved in RNA homeostasis.

### Integrative multi-omics approaches indicate RBM17-mediated splicing prevents NMD of genes required for leukemic growth

**Fig. 4:**
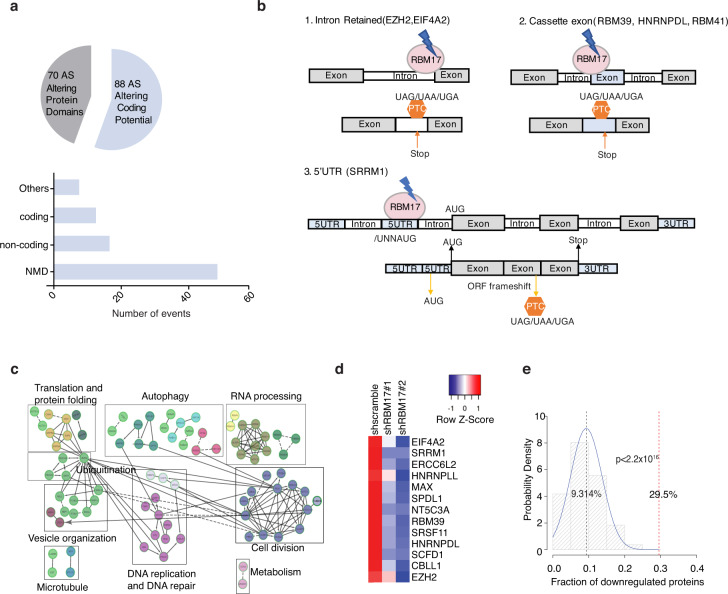
RBM17 knockdown leads to the production of NMD sensitive transcripts. **a** Pie chart distribution of predicted protein consequences, including changes in “protein domain” and “coding potential”. Bottom bar plot indicates the distribution of alternative splicing events predicted to lead to coding potential changes in shRBM17 groups. **b** Depiction of (**1**) RBM17 binding to intronic regions of *EZH* and *EIF4A2*, and resultant promotion of their retention and PTC introduction following knockdown of *RBM17*; (**2**) RBM17 binding to the cassette exon of *RBM39*, *HNRNPDL* and *RBM41* leading to cassette exon inclusion and introduction of PTCs post-RBM17 knockdown; (**3**) RBM17 binding to exon belonging to 5’UTR of *SRRM1* and subsequent inclusion of this exon that contain alternative start codon upon *RBM17* knockdown, inducing ORF frameshift and PTC. **c** Cytoscape network analysis of proteins significantly deregulated by *RBM17* knockdown in K562 cells. **d** Heat map of protein expression fold change of 13 NMD-sensitive transcripts with and without *RBM17* knockdown. **e** Bootstrapping analysis of 44 proteins from 8825 total proteins identified from the *RBM17* knockdown proteome. *P* value was calculated using two-tailed Student’s *t* test.

Analogous to genetic mutations, inclusion or exclusion of certain exons or introns can change the reading frame, which would potentially affect conserved regions of the coded protein structure or have deleterious effect on subsequent mRNA translation. To systematically analyze the potential functional links of RBM17-affected splicing events in AML, we applied a published bioinformatics pipeline to predict effects on the corresponding protein upon *RBM17* depletion^30^. Through this analysis we identified 88 splicing events yielding changes that would alter a transcript from one coding for a functional protein to one marked for nonsense-mediated decay or to a processed transcript that will not yield a protein product (termed “Altering Coding Potential” changes) and 70 splicing changes that would result in a protein being made but having complete or partial loss of functional domains (termed “Altering Protein Domain” changes) (Fig. 4a, Supplementary Data 5). Intriguingly, 13.3% (21/158) of these splicing events cause a complete or partial loss of well annotated protein domains, while 32.9% (52/158) of the splicing events are predicted to produce nonsense-mediated decay (NMD) sensitive transcripts mainly due to the inclusion of poison exons, and the formation of premature termination codons (PTCs) (Fig. 4a). By overlapping the eCLIP-seq dataset and these 52 NMD sensitive transcripts, we identified 6 alternatively spliced transcripts that are predicted to be both bound and regulated by RBM17 (Fig. 4b). It is particularly striking that, within these 6 direct splicing targets of RBM17 are *EZH2* (enhancer of zeste 2 polycomb repressive complex 2 subunit)^31,32^, *RBM39* (RNA binding motif protein 39)^20,21,33^ and *HNRNPDL* (heterogeneous nuclear ribonucleoprotein D like)^34^, all factors that have known important roles in cancer stem cell self-renewal and myeloid malignancy. Together, these studies suggest that *RBM17* knockdown leads to NMD of genes involved in leukemia propagation.

To validate whether these RBM17-mediated NMD-sensitive splicing events cause corresponding protein downregulations, we applied LC-MS proteomics to characterize proteome changes after *RBM17* knockdown in the K562 cells. At day 5 after lentiviral transduction for *RBM17* knockdown, we identified 1157 proteins with significant changes (FDR < 0.1, FC < 0.9 or >1.1) (Supplementary Data 6). GO analysis showed that these proteins downstream of *RBM17* knockdown are enriched in clusters of functional networks representing cell division, RNA processing, autophagy, DNA replication and DNA repair, translation and protein folding and vesicle organization (Fig. 4c). Importantly, when we overlap RBM17-mediated NMD-sensitive splicing targets with the proteomics dataset, among 44 transcripts with measured protein expression values (the other 8 genes were not detected in the proteomics dataset), we demonstrated that 29.5% (13/44) of them are downregulated by *RBM17* knockdown at their protein levels (Fig. 4d). GO analysis of these 13 proteins downregulated by *RBM17* knockdown through potential NMD further revealed a cluster of RNA processing and RNA splicing genes, further supporting that RBM17 plays an important role in controlling RNA processing protein networks that both directly and indirectly influences cancer stem cell biology. We next performed bootstrapping analysis by taking random sets (repeated 10,000 times) of 44 proteins out of the list of 8825 proteins identified in our shRBM17 versus shscramble proteomics experiments to calculate the percentage of proteins that were down-regulated (Mean FC < 0.9, FDR < 0.1) within these 10,000 randomly picked 44-protein sets. Strikingly, the mean percentage of down regulated proteins from randomly-picked 10,000 runs was 9.3%, which is significantly lower than 29.5% observed for predicted NMD sensitive transcripts (*p* < 2.2×10^-16^) (Fig. 4e), suggesting that RBM17 indeed controls protein expression through regulating alternative splicing coupled with NMD.

### RBM17 suppresses EIF4A2 poison exon inclusion

**Fig. 5:**
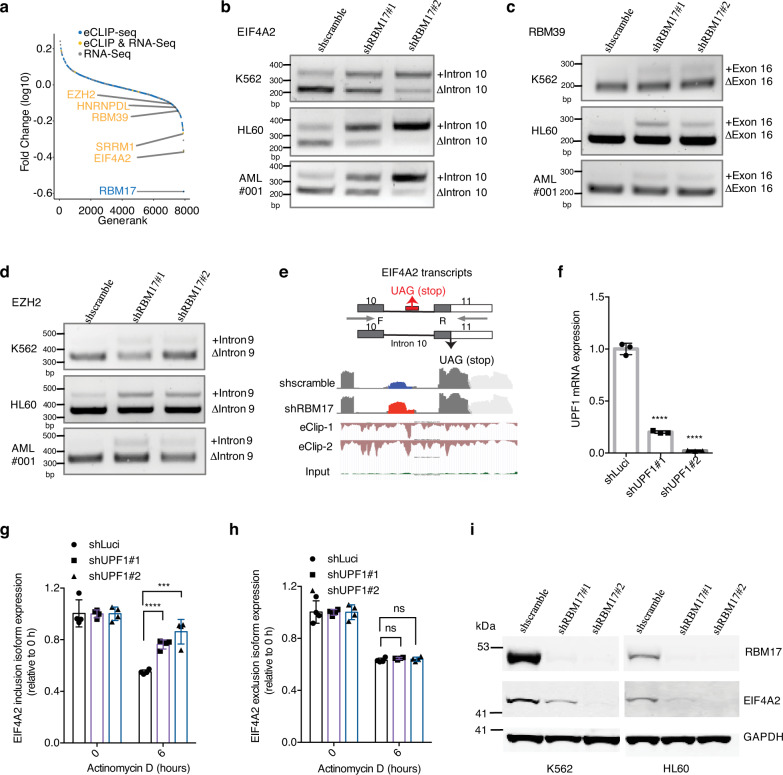
RBM17 suppresses EIF4A2 poison exon inclusion. **a** Ranked protein expression change curve 5 days after transduction with shRNAs against *RBM17* in K562 cells. Protein names that also have significant RBM17 binding with their transcripts (eCLIP-seq) and have significant alternative splicing changes after *RBM17* knockdown (at day 5 by RNA-seq) are displayed (colored in yellow). **b**–**d** RT-PCR validation of *EIF4A2* (**b**), *RBM39* (**c**) and *EZH2* (**d**) variants using RNA extracted from K562, HL60 and primary AML cells with and without *RBM17* knockdown. *n* = 3 independent experiments. **e** Upper: Diagram of *EIF4A2* splicing variants and the primers for RT-PCR detection of cryptic exon inclusion. Bottom: IGV plot of RNA-seq data illustrating the *EIF4A2* cryptic exon promoted by *RBM17* knockdown and anti-RBM17 eCLIP-seq tracks. **f** qPCR quantification of *UPF1* knockdown in K562 cells. *n* = 3, mean ± SD, two-tailed Student’s *t* test. **g** *EIF4A2* intron 10-included isoform in K562 cells with and without *UPF1* knockdown and actinomycin D treatment. *n* = 4, mean ± SD, two-tailed Student’s *t* test. **h** *EIF4A2* intron 10-excluded isoform in K562 cells with and without *UPF1* knockdown and actinomycin D treatment. *n* = 4, mean ± SD, two-tailed Student’s *t* test. **i** Western blot detection of *EIF4A2* after *RBM17* knockdown in K562 and HL60 cell lines. *n* = 3 independent experiments. ***P < 0.001, ****P < 0.0001. Source data are provided as a Source Data file.

Our integrative multi-omics RNA-interactome, transcriptome and proteome profiling analyses revealed that *EIF4A2* (eukaryotic translation initiation factor 4A2), *SRRM1* (serine and arginine repetitive matrix 1), *RBM39*, *HNRNPDL* and *EZH2* are direct NMD-sensitive splicing targets of RBM17 in leukemic cells (Fig. 5a). Using isoform-specific RT-PCR in AML cell lines and primary AML samples, we then validated that RBM17 knockdown promoted inclusion of the poison exons/introns for each of the abovementioned targets (Fig. 5b–d, Supplementary Fig. 4a, b). In addition, for *EIF4A2*, *SRRM1* and *HNRNPDL*, whose poison exon/intron-included isoforms are basally present in AML cells, overexpression of RBM17 inhibited the inclusion of their poison exons/introns (Supplementary Fig. 4c). Among these direct NMD-sensitive splicing targets, the protein level of EIF4A2 is mostly differentially expressed upon RBM17 knockdown, therefore we aimed next to explore its function as a potential effector of RBM17 in AML. RBM17 binds to EIF4A2 intron 10 and *RBM17* knockdown promotes the inclusion of a proximal “poison” cassette exon (*chr3:186788310-186788416*), which is associated with strong depletion of EIF4A2 protein (Fig. 5e). This 107-bp cryptic exon and its flanking intronic sequences are highly conserved across vertebrates (Supplementary Fig. 4d), strongly suggesting that it has a regulatory function^35^. Next, to confirm NMD-sensitivity of this *EIF4A2* transcript variant that includes the poison exon, we tracked its mRNA decay level after actinomycin D-induced transcription-halt in cells with depletion of UPF1, a protein required for NMD. We demonstrated that the mRNA level of the poison exon-included *EIF4A2* variant dropped less with *UPF1* knockdown compared to shLuci control (Fig. 5f, g). Conversely, the mRNA level of the poison exon-skipped *EIF4A2* variant dropped similarly following *UPF1* knockdown as compared to control (Fig. 5h). These results confirmed that RBM17 suppressed the *EIF4A2* poison exon inclusion event, which would otherwise trigger EIF4A2 degradation through NMD. Lastly, we validated that *RBM17* knockdown in a panel of AML cell lines consistently reduced EIF4A2 protein expression (Fig. 5i). Taken together, our results demonstrate that RBM17 is required for the generation of productive protein-coding transcripts of several pro-leukemic factors and identify EIF4A2 as a bona fide direct downstream target of RBM17.

### EIF4A2 is elevated in human LSC and is required for leukemogenesis

**Fig. 6:**
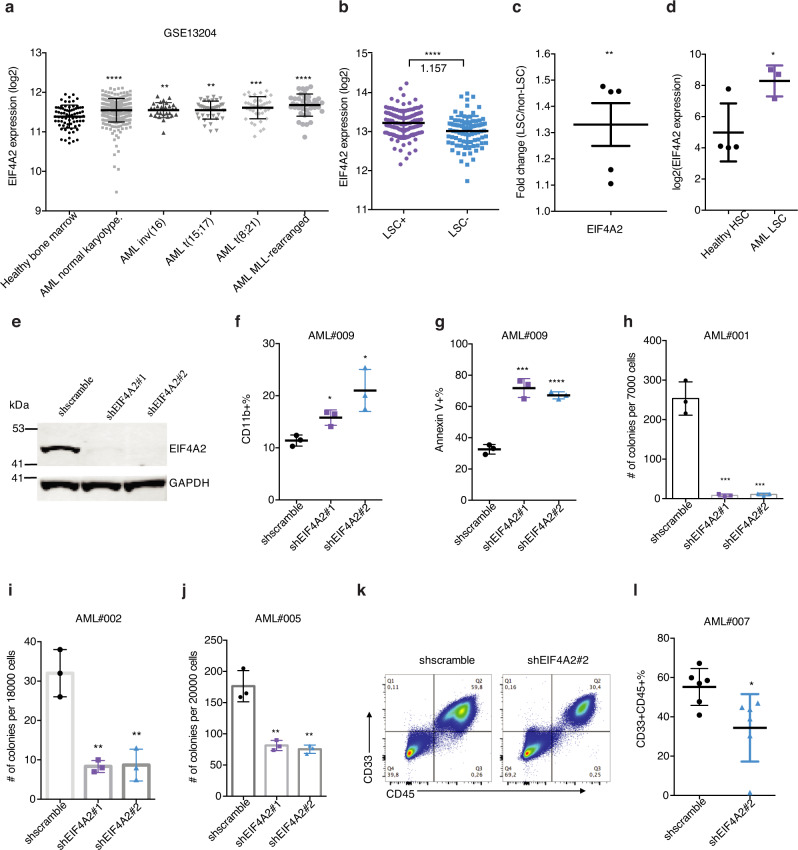
EIF4A2 is required for sustaining primitive leukemic cell fitness. **a** *EIF4A2* mRNA expression in different subtypes of AML as compared to healthy bone marrow control, on the basis of data from Microarray Innovations in Leukemia (MILE) study (GSE13204). Data are presented as mean ± SD, two-tailed Student’s *t* test. **b** *EIF4A2* transcript level in LSC-enriched (*n* = 138) vs LSC-depleted (*n* = 89) subsets from 78 AML patient samples (GSE76008). Average fold change of *EIF4A2* expression in LSC-enriched over LSC-depleted subsets is indicated in the figure. Data are presented as mean ± SD, two-tailed Student’s *t* test. **c** EIF4A2 protein level as assessed proteomics analysis in functionally defined LSC and non-LSC populations from 5 AML patient samples (PXD008307). Data are presented as mean ± SEM, two-tailed Student’s *t* test. **d** Gene expression data (GSE35008) from sorted AML bone marrow samples were compared with data from healthy controls and revealed significantly increased *EIF4A2* expression in AML LT-HSCs (Lin^-^CD34 + CD38^-^CD90 + , AML with normal karyotype, *n* = 3) compared with healthy control (*n* = 4). Data are presented as mean ± SD, two-tailed Student’s *t* test. **e** WB validation of *EIF4A2* knockdown in GFP + transduced HL60 cells. **f**, **g** Flow cytometric evaluation of myeloid differentiation (**f**) and apoptosis (**g**) following *EIF4A2* knockdown in primary AML cells. *n* = 3, mean ± SD, two-tailed Student’s *t* test. **h**–**j** Colony formation capacity following *EIF4A2* knockdown in 3 AML samples. *n* = 3, mean ± SD, two-tailed Student’s *t* test. **k**, **l** Representative flow cytometry plots (k) and quantification (l) of AML engraftment (#007) after 8 weeks in bone marrow. *n* = 6 for each group. Shown is the percentage of human (CD45^+^) myeloid cells (CD33^+^) found in bone marrow. Data are presented as mean ± SD, two-tailed Student’s *t* test. Source data are provided as a Source Data file.

*EIF4A2* encodes an ATP-dependent RNA helicase, which is a subunit of the EIF4F complex involved in ribosome binding to mRNA substrates and scanning for the initiator codon^36^. Intriguingly, through data analysis of the MILE study dataset (GSE13204)^37,38^, we found that *EIF4A2* mRNA was more highly expressed in five subtypes of AML than in normal monocytes (Fig. 6a). Importantly, in the context of LSCs, just as in the case of RBM17, EIF4A2 is preferentially expressed in LSC-enriched cell fractions compared to LSC-devoid fractions from AML patients at both mRNA and protein levels (Fig. 6b, c). Consistent with *RBM17* expression in LT-HSC, *EIF4A2* is also more highly expressed in AML cells with a primitive immunophenotype (Lin-CD34^+^CD38^-^CD90^+^) compared to normal control HSCs (Fig. 6d). Given our demonstration that EIF4A2 is downstream of RBM17, we next aimed to explore the effect of *EIF4A2* knockdown on primitive AML cell function. Towards this end, we depleted *EIF4A2* in AML cell lines and in primary AML samples using two independent shRNAs (#1 and #2) (Fig. 6e). Strikingly, knockdown of *EIF4A2* significantly inhibited AML cell growth (Supplementary Fig. 5a, b), induced myeloid differentiation (Fig. 6f, Supplementary Fig. 5c–d) and resulted in increased cell apoptosis (Fig. 6g, Supplementary Fig. 5e–g) as compared to a shscramble control. In addition, depletion of *EIF4A2* in three primary AML samples significantly inhibited their colony-forming abilities (Fig. 6h–j). Next, to assess the effect of *EIF4A2* knockdown on LSC survival in vivo, we measured engraftment capacity of AML cells following *EIF4A2* depletion. We observed that knockdown of *EIF4A2* in primary AML significantly inhibited engraftment potential (Fig. 6k–l). Together, these data together indicate that EIF4A2 supports the proliferation, survival and undifferentiated state of AML cells.

### EIF4A2 overexpression partially rescues RBM17 knockdown-mediated phenotypes in AML cells

**Fig. 7:**
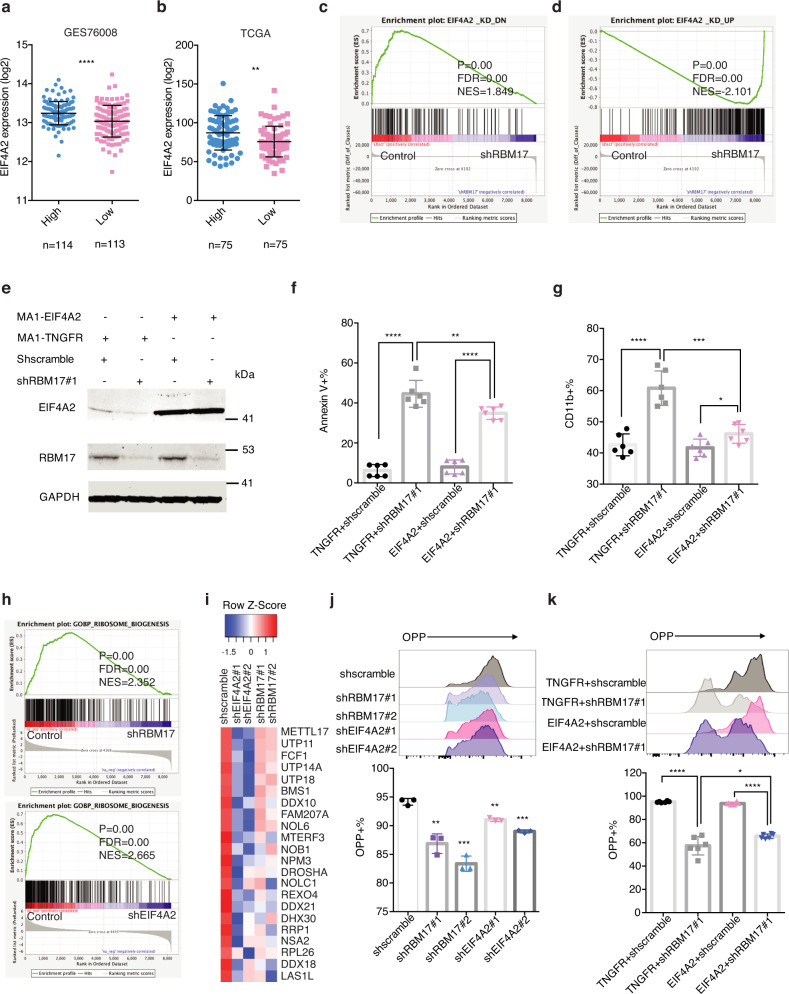
EIF4A2 overexpression partially rescues the RBM17 knockdown phenotype in AML cells. **a**, **b** Correlation analysis between *RBM17* and *EIF4A2* mRNA levels using the above microarray data about functionally defined 138 LSC-enriched and 89 non-LSC populations (*P* < 0.0001) (**a**) and TCGA dataset (*P* = 0.0013) (**b**). AML patient samples were ranked based on *RBM17* expression, the population above the median value of *RBM17* expression level was defined as ‘high’ *RBM17* expression, while the population below the median value of *RBM17* expression level was defined as ‘low’ *RBM17* expression. Data are presented as mean ± SD, two-tailed Student’s *t* test. **c**, **d** GSEA enrichment plots showing *RBM17* knockdown in K562 cells leads to downregulation of the *EIF4A2*_KD_DN gene set and upregulation of the *EIF4A2*_KD_UP gene set. The significance of NES was calculated using Kolmogorov-Smirnov statistics. **e** WB imag**e**s showing expression of RBM17 and EIF4A2 in HL60 cells engineered to co-express TNGFR or EIF4A2 with and without *RBM17* knockdown. *n* = 3 indepe*n*dent experiments. **f**, **g** Flow cytometry analysis of AnnexinV (**f**) and myeloid differentiation (**g**) in HL60 cells on day 8 following co-expression of TNGFR or EIF4A2 and knockdown of control scramble or *RBM17*. Data are presented as mean ± SD, *n* = 6. **h** GSEA enrichment plots showing *EIF4A2* and *RBM17* knockdown in K562 cells leads to downregulation of the GO ribosome biogenesis gene set. The significance of NES was calculated using Kolmogorov-Smirnov statistics. **i** Heat map showing downregulated proteins from the ribosome biogenesis gene set induced by *EIF4A2* and *RBM17* knockdown. **j** Representative histogram and quantification of flow cytometric detection of op-puro incorporation in HL60 cells on day7 following *EIF4A2*/*RBM17* knockdown. Data shown as mean ± SD, *n* = 3, two-tailed Student’s *t* test. **k** Representative histogram and quantification of flow cytometric detection of op-puro incorporation in HL60 cells on day10 following simultaneous expression of TNGFR or EIF4A2 with or without *RBM17* knockdown. Data shown as mean ± SD, *n* = 6, two-tailed Student’s *t* test. Source data are provided as a Source Data file.

Through correlation analysis, we found that RBM17 supports higher expression of *EIF4A2* in two different AML patient datasets (Fig. 7a, b). To test the extent that the downstream effects of *RBM17* knockdown are shared upon *EIF4A2* knockdown, we first performed LC-MS proteomics to characterize proteome changes induced by *EIF4A2* knockdown in K562 cells. We identified a list of significantly downregulated and upregulated proteins induced by *EIF4A2* knockdown (Supplementary Data 7). Interestingly, these two gene sets are significantly enriched in proteins modulated downstream of *RBM17* knockdown as we observed in K562 cells (Fig. 7c, d), suggesting that *EIF4A2* knockdown indeed largely recapitulates the biological effects of *RBM17* knockdown in AML. Given the significant link between EIF4A2 and RBM17 in AML, we next tested whether restoring EIF4A2 in a form impervious to splicing, could rescue any biological effects caused by *RBM17* knockdown. Specifically, we infected HL60 cells with lentiviruses co-expressing a scramble or *RBM17* targeting hairpin with either a truncated NGFR (TNGFR) control cDNA or the *EIF4A2* cDNA (shscramble + TNGFR, shscramble+EIF4A2, shRBM17#1+TNGFR, shRBM17#1 + EIF4A2) (Fig. 7e). We found that overexpression of EIF4A2 partially reversed the adverse effects of *RBM17* knockdown on AML cell apoptosis, cell growth (Fig. 7f, Supplementary Fig. 5a) and partially rescued AML cell differentiation induced by *RBM17* knockdown (Fig. 7g).

To investigate downstream pathways shared between *RBM17* knockdown and *EIF4A2* knockdown, we overlapped differentially expressed proteins identified from *RBM17* or *EIF4A2* knockdown (FDR < 0.1, FC < 0.9 or FC > 1.1). We found that 166 proteins were downregulated and 151 proteins upregulated by *RBM17* and *EIF4A2* loss (Supplementary Fig. 6b). GO analysis showed that proteins downregulated by *RBM17* and *EIF4A2* knockdown are significantly enriched in a variety of pathways, such as DNA replication, DNA repair, cell division, RNA processing, RNA secondary structure unwinding and covalent chromatin modification (Supplementary Fig. 6c). Interestingly, our subsequent GSEA analysis revealed that both *RBM17* and *EIF4A2* knockdown in K562 cells strongly alter expression of proteins enriched in ribosome biogenesis-related gene sets (Fig. 7h), and several known translation-related factors (Fig. 7i, Supplementary Data 8), indicating that downregulation of RBM17 and EIF4A2 may both affect mRNA translation in leukemic cells. To confirm this finding, we performed an O-propargyl-puromycin (OPP) based protein synthesis assay and detected significantly decreased mRNA translation activity in both *RBM17*- and *EIF4A2*-knockdown AML cells, respectively (Fig. 7j). Moreover, we demonstrated that elevation of EIF4A2 level in *RBM17*-knockdown AML cells partially rescued the protein synthesis rate (Fig. 7k). These results together suggest that RBM17 inhibits AML apoptosis and differentiation and supports mRNA translation at least partially through enforcing the expression of an NMD-resistant transcript variant and promoting the expression of EIF4A2 protein in human leukemic cells.

## Discussion

RBM17, also known as splicing factor 45 kDa (SPF45), was originally identified as a component of the spliceosome complex. It co-localizes with SR proteins in nuclear speckles and regulates the second step of pre-mRNA splicing by selecting alternative AG splice acceptor sites^39^. RBM17 protein expression is limited in normal tissues and is greatly increased (5–10 fold) in solid tumors of the bladder, lung, colon, breast, ovary, pancreas, and prostate^40^. In addition, RBM17 has been previously linked to cancer chemotherapy resistance in breast and ovarian cancer cell lines through unspecified mechanisms^41,42^. However, the role of RBM17 in AML has not been explored. Interestingly, using proteomics, we previously found that RBM17 is upregulated in human pluripotent stem cells (PSCs) compared to terminally differentiated fibroblasts and is required to support PSC self-renewal^43^, a core feature shared in both normal and cancer stem cells. Our work identified RBM17 as the sole mRNA splicing factor that is both upregulated in LSC-enriched cell fractions and is significantly associated with poor prognosis of AML patients. Mechanisms governing similar LSC-enhanced expression changes extend from dysregulation of epigenetic modifiers to altered expression of activating transcription factors^44,45^, but myriad other explanations are possible and the exact means by which RBM17’s expression is controlled in LSCs will therefore be an intriguing area for future study. Through analyzing differentially spliced mRNA transcripts upon *RBM17* knockdown in K562 cells, we also noted that many splicing events affected by *RBM17* knockdown have previously been found abnormally spliced in secondary AML (sAML) LSCs compared to aged normal hematopoietic progenitor cells (HPCs)^15^, including transcription related factors TAF6, NFE2, TBL1XR1, CNOT2, MGA, COMMD3, SUPT5H, and RNA processing proteins RBM39, SF1 and EIF4H. These results highlight the likelihood that RBM17 contributes to the aberrant AS program found in the primitive cells that drive the disease. Through the use of gold-standard in vivo repopulation assays with primary AML samples, we demonstrated that *RBM17* depletion impairs the function of disease- and relapse-initiating primitive AML cell compartment. Together these data position RBM17 as a leukemic stem cell regulator whose expression and targeting may have important implications in the diagnosis and treatment of malignant hematopoiesis.

A previous study of splicing in mouse neurons showed that RBM17 normally represses the splicing of cryptic junctions and its loss leads to the inclusion of intronic elements in mature transcripts^46^. More recently, siRNA screening against 154 human nuclear proteins identified that RBM17 is essential in the efficient splicing of many short introns and such splicing regulation is determined by the length of the poly-pyrimidine tract (PPT) followed by the 3’ splice site^47^. Exonization of intronic coding cassettes normally creates frameshifts or introduces PTCs^48,49^. Our integrative multi-omics analysis of RBM17 uncovered that *RBM17* depletion promotes inclusion of poison cassette exons or introns for a number of pro-leukemic factors and leads to their NMD-mediated mRNA degradation and subsequent protein-level downregulation. Our results identified the pro-leukemic factors RBM39, EZH2, and HNRNPDL as direct RBM17 mRNA-binding targets with these interactions serving to preserve their protein levels through exclusion of poison exons. A recent study using CRISPR/Cas9 screening demonstrated that complete loss of *RBM39* suppresses AML growth both in vitro and in vivo, while pharmacologic RBM39 degradation results in broad anti-leukemic effects and preferential lethality of spliceosomal mutant AML^20^. In the same study it was also found that *RBM39* loss affects splicing of mRNAs related to RNA-splicing, export, and metablism^20^. Similarly, EZH2 is an important regulator of normal and malignant hematopoiesis^50^, while HNRNPDL overexpression in CML cells has been shown to induce leukemia in vivo^34^. These findings indicate the possibility that *RBM17* knockdown-induced inhibition of these factors contributed to anti-leukemic effects. Our work has therefore provided mechanistic insights into essential AML molecular circuitry by uncovering that the elevated expression of RBM17 serves to selectively represses the formation of PTC containing mRNAs required for supporting LSC function. Previous studies have found the phosphorylation of RBM17 regulated by mitogen-activated protein kinase (MAPK)^42^ and Cdc2-like kinase 1(Clk1)^51^ affects its alternative splicing site utilization highlighting the possibility that selective small molecule Clk inhibitors^52,53^ may offer a strategy for targeted RBM17 interference as an AML therapy.

Interestingly, our proteomics data showed that *RBM17* knockdown causes downregulation of its known spliceosome interactors CHERP and U2SURP (Supplementary Data 6). This is in line with a previous study in human HEK293T cells showing that RBM17, CHERP and U2SURP reciprocally regulate each other’s expression level and share downstream splicing targets enriched for RNA-binding proteins^29^. We speculate that RBM17, and the spliceosome complex it interacts with, collectively block the usage of cryptic splice sites around cassette exons or introns containing PTCs and skip their inclusions. Furthermore, our bootstrapping analysis indicated that *RBM17* knockdown leads to downstream gene expression inhibition through splicing-coupled NMD, suggesting that RBM17 preferentially regulates splicing of mRNAs containing PTCs. Exploration of the mechanisms through which RBM17 mediates its specific splicing of NMD-sensitive transcripts will be of interest to pursue in future studies.

In the present work, we showed that RBM17 represses the inclusion of poison intron 10 in the *EIF4A2* pre-mRNA, which prevents *EIF4A2* mRNA NMD and promotes its downstream protein synthesis. Therefore, EIF4A2 represents a potential therapeutic target for AML and intersection between splicing and translation control. Previously, Sadlish et al found that the natural compound rocaglamides stabilizes EIF4A-RNA interactions and interferes with the assembly of the EIF4F complex, thereby blocking translation initiation^54^. More recently, Callahan and colleagues showed that rocaglamide is able to preferentially kill functionally defined LSC, but relatively spares normal HSPCs through mechanisms beyond simply inhibiting translation initiation^55^. Since rocaglamide does not distinguish EIF4A family members, it has not been clear which member of the EIF4A family underlies the anti-leukemia effects of the compound. Our work clearly demonstrates that EIF4A2 is more highly expressed in LSCs and is required to support the proliferation, survival and undifferentiated state of AML cells, indicating the RBM17/EIF4A2 axis we have uncovered is indeed targetable for AML treatment and that the inhibitory nature of rocaglamide on AML function is likely due largely to its effects on EIF4A2. Importantly, a previous comparative EIF4A1 and EIF4A2 RIP-seq study in HEK293 cells also showed that 23% of EIF4A2’s RNA targets are unique^56^. GO analysis of the EIF4A2-specific RNA targets further showed that these genes are involved in transcription, cell migration, cell cycle and positive regulation of GTPase activity. Many of EIF4A2’s unique RNA targets, such as USP6NL (a GTPase-activating protein) and REV1, both of which are significantly upregulated in LSC, are indeed downregulated by EIF4A2 knockdown in K562 cells. Thus, understanding the functional role of the EIF4A2-specific RNA targets in AML and LSC may provide mechanistic support for specifically targeting EIF4A2 and/or its regulated pathways for AML treatment and directing the improvement of drug target sensitivity.

Upregulation of protein synthesis has been described to occur in “pre-leukemic” myelodysplastic (MDS) stem cells^57^. AML stem cells also exhibit increased expression of ribosome pathway genes^58^, indicating the potential role of ribosome biogenesis in the establishment and propagation of cancer stem cells in the blood system. Importantly, both RBM17 and EIF4A2 knockdown inhibited protein synthesis and downregulated at the protein level, the expression of factors enriched in the ribosome biogenesis pathway, suggesting a link between elevated expression of RBM17 along with its downstream target EIF4A2 and protein synthesis activation in primitive AML cells. Our EIF4A2 rescue experiments support the concept that *EIF4A2* inhibition is necessary for the shRBM17-induced apoptosis and contributes to shRBM17-induced myeloid differentiation and translation inhibition. Interestingly, our proteomics analysis showed that *RBM17* knockdown also led to downregulation of other translation-related factors including UBA52, EIF4H and EIF3B (Supplementary Data 6), the collective loss of which may synergize with that of EIF4A2 to further solidify the translation inhibitory effects induced by RBM17 loss.

**Fig. 8:**
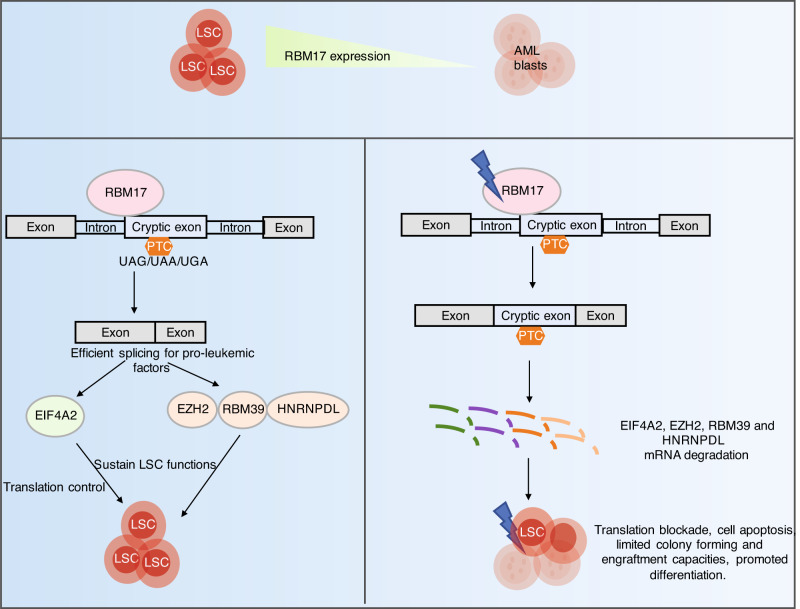
Schematic model depicting the role of RBM17 in primitive AML cells. RBM17 is abnormally higher expressed in the most primitive cell fractions of AML compared to AML blasts, which contributes to efficient splicing of many pro-leukemic factors EZH2, RBM39 and HNRNPDL, along with EIF4A2 that functions in translation control, to sustain LSC functions. Knockdown of RBM17 promotes inclusions of cryptic exons or introns into mRNAs of these pro-leukemic factors, leading to their mRNA degradations due to NMD and consequently resulting in translation blockade, cell apoptosis, limited colony-forming and engraftment capacities, and promoted differentiation in primitive AML cells.

In conclusion, human AML stem and progenitor cells express abnormally high levels of RBM17 to ultimately enforce NMD-escape for a number of key pro-LSC transcripts (Fig. 8). In particular, we place this mechanism of RBM17-directed control upstream of the essential process of protein synthesis in LSC and identify inhibition of the RBM17-EIF4A2 axis as a potential therapeutic avenue for AML treatment.

## Methods

### Mice

NOD-*scid*-*IL2Rγc*^*−/−*^ (NSG) (Jackson Laboratory) mice were bred and maintained in the Stem Cell Unit animal barrier facility at McMaster University and University Health Network/Princess Margaret Cancer Centre. All procedures received the approval of the Animal Research Ethics Board at McMaster University, HiREB (Hamilton Integrated Research Ethics Board), the Canadian Counsel on Aminal Care and the University Health Network Animal Care Committee. The housing lights are on a 12 hours on/ 12 hours off cycle (6 am on and 6 pm off everyday), the housing temperature is between 19-21°C and the humidity is 40~60%.

### Primary CB and AML patient samples

All umbilical cord blood (CB) and AML patient samples were obtained as donations with written informed consent and with the approval of the local human subject research ethics board at the University Health Network and McMaster University in accordance with Canadian Tri-Council Policy Statement on the Ethical Conduct for Research Involving Humans (TCPS). AML samples were donated from patients with ages ranging from 27 to 81.5. Patients were aware of the research purpose and were not compensated for their donations. Following Ficoll-Paque separation mononuclear cells were stored in the vapor phase of liquid nitrogen in 10% DMSO, 30% fetal bovine serum (FBS) and 60% Dulbecco’s modified Eagle’s medium (DMEM). Primary samples were thawed in warmed 50% FBS, 48% X-VIVO with 100 µg/ml DNAse (AML) or 100% FBS (CB) prior to using in in vitro and in vivo assays.

### Cell culture, cell lines and flow cytometry

The human promyelocytic leukemic cell line NB4 (DSMZ, ACC 207), myeloblastic cell line HL60 (ATCC, CCL-240), and chronic myelogenous leukemic cell line K562 (ATCC, CCL-243) were cultured in RPMI-1640, supplemented with 10% FBS. HEK 293FT (ATCC, CRL-1573) and LentiX-293T (Clontech/Takara, 632180) cells were cultured in DMEM, supplemented with 10% FBS. OCI-AML8227 patient-derived model was a gift from Dr. Eric Lechman^25^. OCI-AML-8227 cells were cultured in StemSpan SFEM II (Stem Cell Technologies) with Interleukin (IL-3, 10 ng/mL, R&D Systems), stem cell factor (SCF, 50 ng/ml, R&D Systems), Interleukin-6(IL-6, 10 ng/ml, R&D Systems), FMS like tyrosine kinase 3 ligand (FLT3, 50 ng/mL, R&D Systems), Thrombopoietin (TPO, 25 ng/mL, Peprotech), and Granulocyte Colony-Stimulating Factor (G-CSF, 10 ng/mL, Peprotech). OCI-AML-8227 cells were plated 400,000 cells/ 24 well non-adherent culture plate and were split every 6–7 days. Primary AML samples were grown in X-VIVO with 20%BIT, 100 ng/mL human SCF, 100 ng/mL human FLT3, 20 ng/mL human TPO, 20 ng/mL human IL3 and 10 ng/mL human IL3. Human CB derived hematopoietic stem and progenitor cells (HPSCs) were cultured in StemSpan SFEM II (Stem Cell Technologies) with 20 ng/mL human TPO, 100 ng/mL human SCF, 100 ng/mL human FLT3, 20 ng/mL human IL6 and Penicilin-Streptonmycin. All cells were incubated at 37 °C in a humidified atmosphere containing 5% CO2. All flow cytometry analysis was performed using a BD LSRII flow cytometer, BD LSR Fortessa, MACSQuant Analyzer and FlowJo Software (v7.6.5). Cell sorting was performed with MoFlo XDP (Beckman Coulter) and BD FACS Aria III.

### AML and CB transduction

For AML cell lines HL60, K562 and NB4, cells were infected with lentivirus using a multiplicity of infection of 2–10 followed by puromycin selection or 7-AAD^-^ and GFP^+^ or BFP^+^ sorting. For primary AML samples, 1.5 million cells were infected with lentivirus using an MOI of 50 in 24 well ultralow attachment plates with 500ul total growth media, another 500ul of growth media were added 16 hours after infection followed by 7AAD^-^ and GFP^+^ sorting. For CB transduction, flow-sorted Lin^-^CD34^+^ or Lin^-^CD34^+^CD38^-^ cells were prestimulated for 6 hours in StemSpan medium II (StemCell Technologies) supplemented with growth factors. Lentivirus was then added at a MOI of 50 and cells were grown for another 3 days before flow sorting or xenograft study.

### Colony-forming unit assay

Sixteen hours (for primary AML) or 72 h (for Lin^-^CD34^+^ CB) post lentiviral infection, freshly sorted 7- AAD^-^ and GFP^+^ or BFP^+^ (healthy shRNA-expressing) cells were plated in triplicates in Human Methylcellulose Complete Media (ColonyGEL #1102), and grown in 37 °C, 5% CO2 incubator for 10–14 days before imaging and counting. For primary AML, ~7000–30,000 cells per ml were plated. For CB, 600 cells per/ml were plated.

### Xenograft studies

NSG mice were sublethally irradiated (315 cGy) 1 day prior to injection. Pre-validated engrafting AML samples were infected with shRNA for 24 hours in 24-well culture plates at a MOI of 50. Flow sorted Lin^-^CD34^+^CD38^-^ CB cells were infected with shRNA lentivirus for 72 h at a MOI of 50. Post transduction, cells were validated for GFP expression (monitored sample of cells until 3 days after transduction), washed, resuspended in IMDM + 1% FBS. Then 200,000 ~ 1,000,000 AML cells or ~10,000 CB cells were injected with 30ul of IMDM + 1% FBS into the right femur of each recipient mouse, 5 ~ 6 mice were injected per experimental group. 8–12weeks post-transplant, mice were sacrificed and bone marrow from tibias, femurs and pelvis was harvested, crushed with mortar and pestle, filtered and red blood cell lysed using ammonium chloride buffer. Human AML/CB engraftment was analyzed by blocking reconstituted mouse bone marrow with mouse Fc block (BD Biosciences) and human IgG (Sigma), followed by staining with fluorochrome-conjugated antibodies against human CD45-BV-421, CD33-PE, CD34-APC, CD14-APC-H7, CD11b-BV605 (BD Biosciences) for graft analysis.

### Calculation of relative engraftment potential

For AML sample #001 and CB transplant: using flow cytometry, we first measured % of GFP^+^ cells within the 7-AAD^-^ gate to determine % of shRNA-expressing cells injected (Input, %GFP-injected). At the end point, we also measured % of GFP^+^ cells within the 7-AAD^-^ gate and human CD45^+^CD33^+^gate to determine the % of shRNA-expressing cells engrafted (Output, %GFP-engrafted). For each experimental group (e.g. control as *x* and shRBM17 as group *y*), we first calculated engraftment value *x* or *y* using the following formula: *x* or *y* = %GFP- engrafted ÷ %GFP-injected for each mouse. This calculation yielded an array of engraftment values for both control (*x*1, *x*2, *x*3, *x*4, *x*5) and shRBM17 (*y*1, *y*2, *y*3, *y*4, *y*5) groups. To calculate the final engraftment potential score, each engraftment value was normalized by the mean of (*x*1, *x*2, *x*3, *x*4, *x*5) from the control group. These scores were plotted to compare the relative engraftment potential between control and shRBM17.

### Apoptosis and differentiation assays

To measure apoptosis, cells were washed with PBS and incubated with V450-Annexin V (APOAF, sigma) or anti-V450-Annexin V (560506, BD) or Annexin V, Alexa Fluor® 647 Conjugate (A23204, ThermoFisher) and PI or 7-AAD in the Annexin V binding buffer in a reaction volume of 100ul for 15 min according to the manufacture’s instruction analyzed by FACS. To monitor cellular differentiation status, cells were stained with the following antibodies: PE-CD11b (557321, BD), CD11b-BV605 (562721, BD), FITC-CD14 (551376, BD), APC-H7-CD14 (561384, BD), APC-CD15 (551376, BD).

### Intracellular flow cytometry

Primary AML cells and OCI-AML-8227 cells were initially stained with anti-CD34 APC (555824, BD Biosciences) antibody and LIVE/DEAD Fixable Green (L34969, Thermo Fisher) and then fixed with the Cytofix/Cytoperm kit (BD Biosciences) according to the manufacturer’s instructions. Fixed and permeabilized cells were immunostained with anti-RBM17 rabbit antibody (ab204333, Abcam) and detected by Alexa-Fluor 405 goat anti-rabbit IgG(H + L) secondary antibody (Thermo Fisher) through FCAS. For RBM17 knockdown efficiency test, sorted cells were initially stained with LIVE/DEAD Fixable Near-IR (L34975, Thermo Fisher) for 30 minutes and then fixed with the Cytofix/Cytoperm kit. Fixed and permeabilized cells were immunostained with anti-RBM17 rabbit antibody and detected the same way as described above.

### Cell proliferation assay

Cell proliferation was measured by counting the number of cells. In brief, GFP^+^ cells were sorted and seeded (2×10^4^/ml) into 12-well plates after infection with shRNAs. Cells were stained by trypan blue and then counted by Cell Counter according to the manufacturer’s instructions every two days.

### Gating strategy for flow cytometry analysis

For Annexin V detection assay, cell debris were excluded using FSC and SSC, then doublets were excluded using FSC-A and FSC-H, transduced GFP-positive or BFP positive cells were selected and Annexin V-positive cells were further determined. All other samples were initially gated using the FSC/SSC profile to identify events corresponding to cells and not debris, then singlets were selected by plotting FSC-A versus FSC-H and live cells were subsequently further enriched by gating on LIVE/DEAD Fixable Green/NearIR –negative or 7-AAD-negative cells.

1. For RBM17 intracellular flow cytometry, within live cell population, CD34-positive and CD34-negative cells were gated based on unstained controls. Then RBM17-positive and RBM17-negative cells were furthered gated based on unstained cells + Alexa-Fluor 405 goat anti-rabbit IgG(H + L) secondary antibody control within CD34-positive and CD34-negative cells.
2. For in vitro immunophenotyping assays, transduced GFP-positive or BFP-positive cells were selected within live cell population and then CD14, CD15, CD11b positivity were determined with gates set relative to unstained controls. The gating strategy for in vivo immunophenotyping analyses of grafts from primary AML transplanted mice were described in the above “Calculation of relative engraftment potential” method section.

### RNA extraction, qRT-PCR and isoform specific RT-PCR

Total cellular RNA was isolated with Trizol LS reagent (Invitrogen) according to the manufacturer’s instructions and cDNA was synthesized using qScript cDNA Synthesis Kit (Quanta Biosciences, #95047) or LunaScript RT SuperMix Kit (New England BioLabs, #E3010L). For qRT-PCR, samples were prepared with iTaq Universal SYBR Green Supermix (Bio-Rad) and ran as described by the manufacturer, GAPDH or 18 S rRNA was used as internal control. qPCR primer sequences are listed in Supplementary Table 5. For isoform specific RT-PCR, endogenous splicing products of *EIF4A2, RBM39, EZH2, HNRNPDL, SRRM1, MADD, MED24, GSK3β, MPZL1, ZDHHC3, PTPRC, H2AFY, MRPS18C, ZFYVE19, C4orf33, USP33 and AP2B1* genes were analyzed by PCR using the specific primer sets (Supplementary Table 3). The PCR products were then detected using 1.5 ~ 2% agarose gel. All the experiments were repeated 3 ~ 6 times independently.

### Protein lysates preparation and Western blot

Cells were collected and then spalled by lysis buffer. Protein concentration were measured by micro BCA^TM^ protein assay kit. After normalization, equal amount of proteins within 1× LDS loading buffer containing 10% DTT were boiled for 7 min at 95 °C prior to electrophoresis. Proteins were transferred onto NC membrane, blocked with 5% skim milk in 1x TBST for 1 h at room temperature and then incubate overnight at 4 °C with primary antibodies rabbit-anti-RBM17 (A302-498A, Bethyl, 1:1000), anti-EIF4A2 (NBP2-24612SS, Novus Biologicals, 1:1000), rabbit-anti-GAPDH (2118 S, New England BioLabs, 1:2000). Following membrane washing, secondary antibodies IRDye 680RD Goat anti-Rabbit IgG (LIC-925-68071, Mandel Scientific) or IRDye 800CW Goat anti-Rabbit IgG (LIC-925-32211, Mandel Scientific) was added with 1:8000 dilution for 1 h at room temperature and washed 3 times with 1x TBST, then membranes were imaged with the Odyssey Classic Imager (Li-COR Biosciences). RBM17 IP-western blot was probed with a rabbit anti-RBM17 primary antibody (A302-498A, Bethyl, 1:1000) and mouse anti-rabbit IgG (light-chain specific) secondary antibody (45262 S, New England BioLabs, 1:2000) followed by a third IRDye® 800CW Donkey anti-Mouse IgG (H + L) antibody (LIC-92632212, MandelScientific, 1:8000).

### Generation of recombinant lentivirus

PLKO.1 lentiviral vectors that expressing DNA fragments encoding shRNAs against RBM17 (#1: TRCN0000001203, #2: TRCN0000001201) were purchased form Sigma. The negative control shscramble was purchased from Addgene (#136035). For shRNAs containing a GFP selection marker, puro was replaced by EGFP cloned from MA-1 plasmid. shRNAs oligonucleotides against EIF4A2, UPF1 and negative luciferase control were ligated into PLKO.1 lentiviral vector with a GFP marker by using AgEI and ECORI sites. Human EIF4A2 cDNA was cloned from the plasmid pcDNA3.1 + EIF4A2 myc HIS (Addgene, #71658) and ligated into the MA-1 bi-directional lentivirual expression vector^59^ using BamHI site. Sequences of all oligonucleotides are listed in Supplementary Table 4. Recombinant vectors and packaging plasmids PSPAX2 and PMD2.G were transfected into HEK 293FT or LentiX-293T cells to produce recombinant lentivirus.

### eCLIP-seq preparation

RBM17 eCLIP studies were performed in duplicate according to the published eCLIP-seq experimental procedures^34^. 20 million K562 cells were washed in PBS and UV crosslinked at 400 mJoules/cm^2^ with 254 nm radiation on ice, pelleted and snap frozen. Cells were then lysed in iCLIP lysis buffer (50 mM Tris-HCl pH 7.4, 100 mM NaCl, 1% NP-40, 0.1% SDS, 0.5% sodium deoxycholate, 1:100 Protease Inhibitor Cocktail) and treated with Turbo DNase and RNase I to fragment RNA, and supernatants from lysates were collected for immunoprecipitation. 10 μg of RBM17 antibody mixture (Bethyl A302- 497 A and A302-498A) for each sample was then pre-coupled to 125ul of sheep anti-rabbit Dynabeads (LifeTech), added to lysate and incubate overnight at 4°C. Prior to washing, 2% of the sample (with beads-RBM17 antibody-cell lysate) was taken as the size match input sample, with the remainder magnetically separated and washed twice with cold High salt wash buffer (50 mM Tris-HCl pH 7.4, 1 M NaCl, 1 mM EDTA, 1% NP-40, 0.1% SDS, 0.5% sodium deoxycholate) and wash buffer (20 mM Tris- HCl pH 7.4, 10 mM MgCl2, 0.2% Tween-20). After RNA adapter ligation, IP-western, eCLIP was performed by excising the area from 50 kDa to 125 kDa for Input and IP samples. Reverse transcription, DNA adapter ligation, and PCR amplification were performed according to the standard eCLIP experimental procedure. Three libraries (Input and RBM17 eCLIP triplicates) were prepared and sent for paired-end 75-bp Illumina sequencing. For RBM17 eCLIP-seq data analysis, Fastq files were run through eCLIP-v0.4.0 pipeline as described previously^60^.

### RNA-seq analyses

RNA-seq data of shRBM17 or Control transduced K562 cells were downloaded from GSE88633 and GSE88047. Data was pre-processed and filtered using standard parameters. Quality control checks were performed on raw RNA-seq data using FastQC (v0.11.5). Adapter contamination and low-quality sequences in the ShRBM17 and Control (duplicate) samples were removed using the tool FastxToolkit. The quality filtered data was processed for uniform read length (73 bp) using the tool trimmomatic v0.38^61^. The pre-processed samples were individually aligned to Human genome (UCSC HG38) with default settings using the transcriptome aligner STAR v 2.7.2b,—outSAMtype BAM SortedByCoordinate). Differentially spliced events were identified in ShRBM17 samples compared to Control using the rMATS tool (v 4.0.1)^62^. rMATS was run in paired-end mode for the 73 bp uniform-length reads. Splice junction annotations for splicing events were used from ensemble GTF file (GRCh38.96). Five types of splicing events i.e., Exon skipping (CE), Intron retention (RI), Mutually exclusive exons (MXE), Alternative 3’ splice site (A3’SS) and Alternative 5’ splice site (A5’SS) were identified by rMATS. The differentially spliced events identified using rMATS were filtered with FDR < 0.1 in each cohort.

Functional switches were identified using bioinformatics pipelines which has been described previously^30^. The scripts were developed in python to identify splicing events which led to possible functional switches. These included coding potential changes (e.g., from transcripts coding for functional proteins in one condition to the transcripts leading to proteins marked for nonsense-mediated decay or processed transcripts without a protein product in the other condition) or the events which lead to changes in protein product due to frameshift, thus, resulting in complete or partial loss of functional domains. The Bioconductor/R packages maser^63^ and drawProteins^64^ were employed for visualizations of the alterative splicing events in transcripts in context of their protein products.

### Proteomic sample preparation

One million K562 cells transduced with shscramble or shRNA (#1, #2) targeting RBM17 were harvested (day 5 after transduction, replicates for each condition), washed three time with ice cold 1×DPBS. Cell pellets we lysed in 200 µl of lysis buffer composing of 8 M urea (Sigma-Aldrich) and 100 mM ammonium bicarbonate (Sigma-Aldrich). Cells were then vortexed using Mini S-2 Vortex Mixer (Fisher Scientific) for ten seconds, followed by ten seconds of incubation on ice. This procedure was repeated six times. The lysate was then centrifuged at 21,000×g for five minutes at 4 °C. Protein reduction was conducted using 5 mM of tris (2-carboxyethyl) phosphine (Sigma-Aldrich) for 45 minutes at 37 °C. Subsequently, 10 mM of iodoacetamide (Sigma-Aldrich) was added for protein alkylation for 45 minutes at room temperature (dark). Following alkylation, cell lysate was diluted five-fold with 100 mM of ammonium bicarbonate to lower urea concentration. Based on protein amount, Sequencing Grade Modified Trypsin (Promega) was then added in (trypsin: protein(w:w) at 1:50) for overnight digestion at 37 °C. Trifluoroacetic acid (Thermo Scientific) was added to reduce pH, and desalting was conducted with SOLA Solid Phase Extraction 2 mg 96-well plates (Thermo Scientific). Peptides were eluted twice using 200 µL 80% Acetonitrile - 0.1% trifluoroacetic acid. Eluted peptides were speed-vacuum dried using Labconco CentriVap Benchtop Vacuum Concentrator (Kansas City, MO).

### Tandem mass tag Six-plex (TMT 6-plex) labeling, liquid chromatography and tandem mass spectrometry (LC/MS/MS)

TMTsixplex Isobaric Label Reagent Set (Thermo Fisher) was resuspended in LC-MS grade anhydrous acetonitrile (Sigma-Aldrich) following manufacturer’s protocol. Briefly, 0.8 mg of TMT reagent was resuspended in 41 µL of acetonitrile and incubated at room temperature for ten minutes. During the incubation, dried peptide samples were resuspended in 100 mM of triethylammonium bicarbonate (TEAB) (Sigma-Aldrich) to 1 µg/µL. Then, TMT reagents were mixed with peptide samples at 4:1 (wt/wt) ratio and incubated at room temperature for one hour. Following incubation, each TMT reaction was quenched with 8 µL of 5% hydroxylamine (Sigma-Aldrich) for 15 minutes at room temperature. Labeled samples were pooled together at equal ratio and then fractionated on a home-made high-pH C18 column (200 µm x 30 cm, packed with Waters BEH130 C18 5 µm resin) into 36 cuts. All cuts were then injected and separated on homemade trap (200 µm × 5 cm, packed with POROS 10R2 C18 10 µm resin) and analytical column (50 µm × 50 cm, packed with Reprosil-Pur 120 C18-AQ 5 µm resin), with 3 hr reverse-phase gradient delivered by a Thermo Fisher Ultimate 3000 RSLCNano UPLC system coupled to a Thermo QExactive HF quadrupole−Orbitrap mass spectrometer. A parent ion scan was performed using a resolving power of 120,000 and then up to the 20 most intense peaks were selected for MS/MS (minimum ion count of 1000 for activation), using higher energy collision induced dissociation (HCD) fragmentation. Dynamic exclusion was activated such that MS/MS of the same m/z (within a range of 10ppm; exclusion list size = 500) detected twice within 5 s were excluded from analysis for 40 s.

### Proteomic data processing and analysis

LC-MS data generated was analyzed against a UniProt human protein database (42,173 entries) for protein identification and quantification by Thermo Proteome Discoverer (v 2.2.0). Identified proteins have at least one unique peptide. The FDR was calculated from the output P values using Benjamini-Hochberg method. The fold change (FC) of normalized protein expression intensities (FC < 0.9 or FC > 1.1) and FDR < 0.1 was used to identify proteins that are differentially abundant and used for downstream integrative analysis.

### Statistical analysis

Sample sizes are indicated in relevant figures. Experiments were repeated 3 ~ 6 times independently. All statistical analysis (except analysis of eCLIP-seq, RNA-seq and proteomic data sets) was performed using GraphPad Prism (GraphPad Software version 6.0). A paired *t*-test was performed for Fig. 1e. Unpaired student t-tests (two-tailed) were performed for all other studies with *p* < 0.05 as the cutoff for statistical significance. Error bars indicate mean ± SD or mean ± SEM.

### Reporting summary

Further information on research design is available in the Nature Research Reporting Summary linked to this article.

## Supplementary information

Supplementary Information

Description of Additional Supplementary Files

Supplementary Data 1

Supplementary Data 2

Supplementary Data 3

Supplementary Data 4

Supplementary Data 5

Supplementary Data 6

Supplementary Data 7

Supplementary Data 8

Reporting Summary

## Data Availability

The eCLIP-seq data generated in this study have been deposited in NCBI’s GEO under GEO Series accession number GSE180955. The mass spectrometry proteomics raw data have been deposited in the ProteomeXchange Consortium via Proteomics Identification (PRIDE)^65^. The accession number of the proteomics data reported in this paper is PRIDE: PXD026780. Gene expression data of 138 xenotransplant defined LSC-enriched and 89 non-LSC subsets from 78 AML were obtained from GEO (GSE76008)^8^. Gene expression data on sorted LT-HSC (most primitive hematopoietic cells) from AML patients and healthy controls were obtained from GEO (GSE35008)^24^. Protein expression of xenotransplant validated LSC-enriched and non-LSC fractions from 6 AML samples were obtained from PRIDE (PXD008307)^66^. Gene set enrichment analysis (GSEA) was performed by compassion of the “RBM17 high” and “RBM17 low” gene expression profile with the published LSC gene signature (GSE76008)^8^. RNA-seq data of shRBM17 or Control transduced K562 cells were downloaded from GEO (GSE88633) and GEO (GSE88047). Gene expression data of normal human hematopoietic cells were obtained from GEO (GSE42519)^26^. Leucegene AML dataset were obtained from GEO (GSE67040)^23^, BeatAML dataset can be accessed through the link https://www.cbioportal.org/, TCGA-LAML dataset can be accessed through the link https://portal.gdc.cancer.gov/projects/TCGA-LAML. Source data are provided with this paper.

## Source data

Source Data files

## Code availability

Bioinformatic pipelines used for protein effects predition has been described and published previously^30^.
