# Supplementary material for: The splicing factor RBM17 drives leukemic stem cell maintenance by evading nonsense-mediated decay of pro-leukemic factors
Source: Nat Commun. 2022 Jul 4;13:3833. doi: 10.1038/s41467-022-31155-0 (PMC9250932; doi:10.1038/s41467-022-31155-0)
Supplement: Supplementary file 1 — Supplementary Information [file 41467_2022_31155_MOESM1_ESM.pdf]

The Splicing Factor RBM17 Drives Leukemic Stem Cell Maintenance by Evading Nonsense-mediated Decay of Pro-Leukemic Factors

Lina Liu[1,2,3,4], Ana Vujovic[3,4], Nandan P. Deshpande[5,6], Shashank Sathe[7], Govardhan Anande[5,6], He Tian Chen[2,4], Joshua Xu[2,4], Mark D. Minden[3,4], Gene W. Yeo[7], Ashwin Unnikrishnan[5,6], Kristin J. Hope[3,4,*], and Yu Lu[1,*]

[1] Department of Medicine, Faculty of Health Sciences, McMaster University, Ontario, Canada
[2] Department of Biochemistry and Biomedical Sciences, Faculty of Health Sciences, McMaster University, Ontario, Canada
[3] Department of Medical Biophysics, University of Toronto, Ontario, Canada
[4] Princess Margaret Cancer Centre, University Health Network, Ontario, Canada
[5] Adult Cancer Program, Lowy Cancer Research Centre, University of New South Wales, Sydney, Australia
[6] Prince of Wales Clinical School, University of New South Wales, Sydney, Australia
[7] Department of Cellular and Molecular Medicine, Stem Cell Program and Institute for Genomic Medicine, University of California at San Diego, California, United States

* Authors contributed equally

Corresponding authors:
Kristin Hope (kristin.hope@uhnresearch.ca)
Yu Lu (yu.lu@mcmaster.ca)

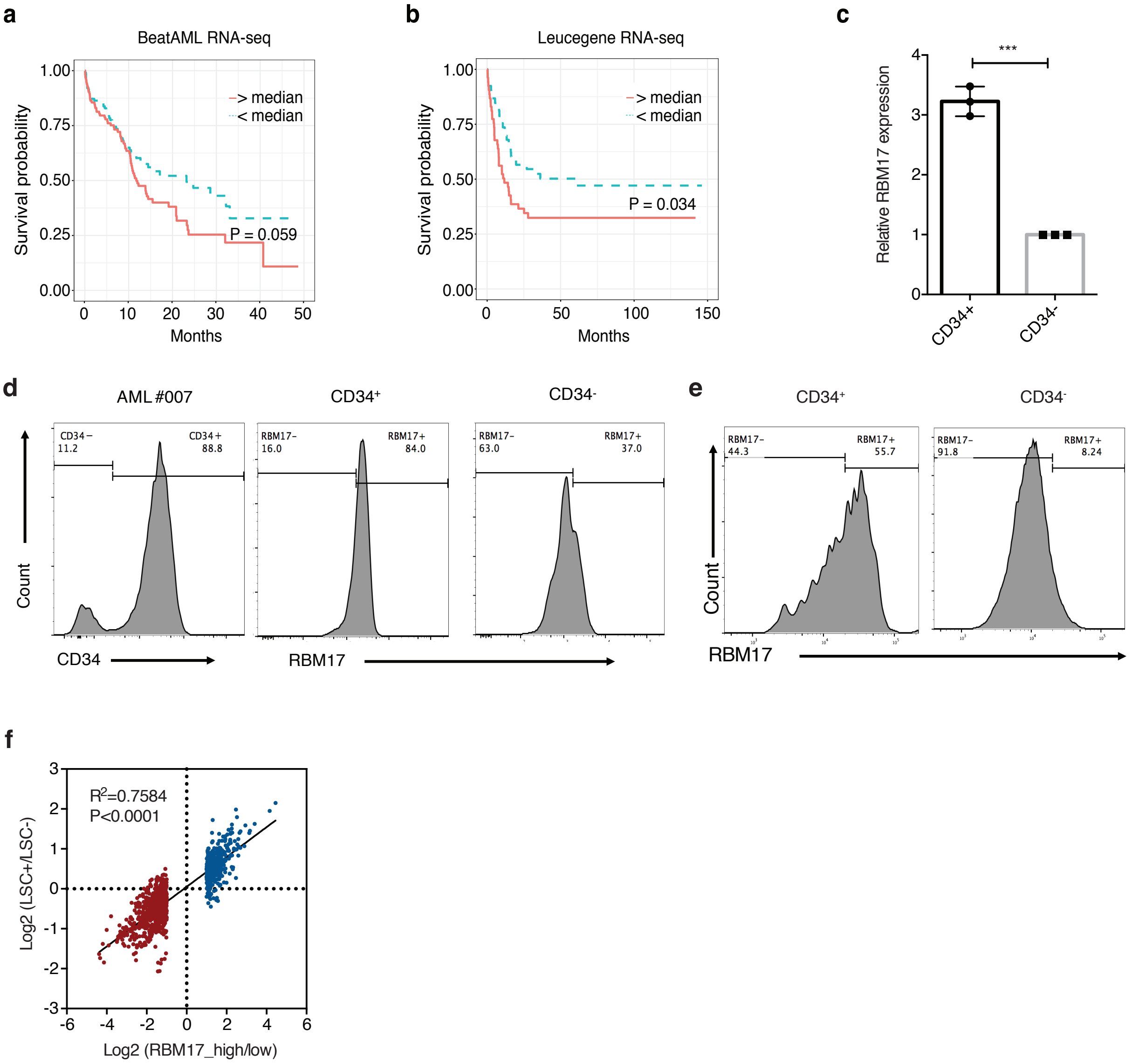

Supplementary Fig. 1: Related to Fig. 1. RBM17 is preferentially expressed in primitive cell fraction of AML. (**a-b**) Kaplan Meier curves showing outcomes of AML patients from the BeatAML dataset (**a**) and Leucegene dataset (**b**) with above vs below median expression of *RBM17*. P value was assessed using log-rank test. (**c**) RBM17 transcript level in the primitive CD34+ vs the committed CD34- subsets in OCI-AML-8227 cells measured by qRT-PCR. *n*=3, mean ± SD, two-tailed Student's *t* test. (**d**) One representative primary AML sample (#007) with intracellular flow cytometry plots showing expression profile of CD34 (left) and percentage of RBM17+ cells in CD34+ and CD34- fractions of this AML sample (right). (**e**) Intracellular flow cytometry plots showing expression profile of RBM17+ cells in CD34+ and CD34- fractions of OCI-AML-8227 cells. (**f**) Correlation analysis showing fold changes (log2) of 832 differential expressed genes identified from *RBM17*-higher AML cases versus *RBM17*-low cases in *RBM17*-high/low AML patients (x axis) and in LSC/non-LSC subsets of 78 AML patients (y axis). Correlation coefficient R squared and P value were calculated based on Pearson's correlation test, two-tailed.

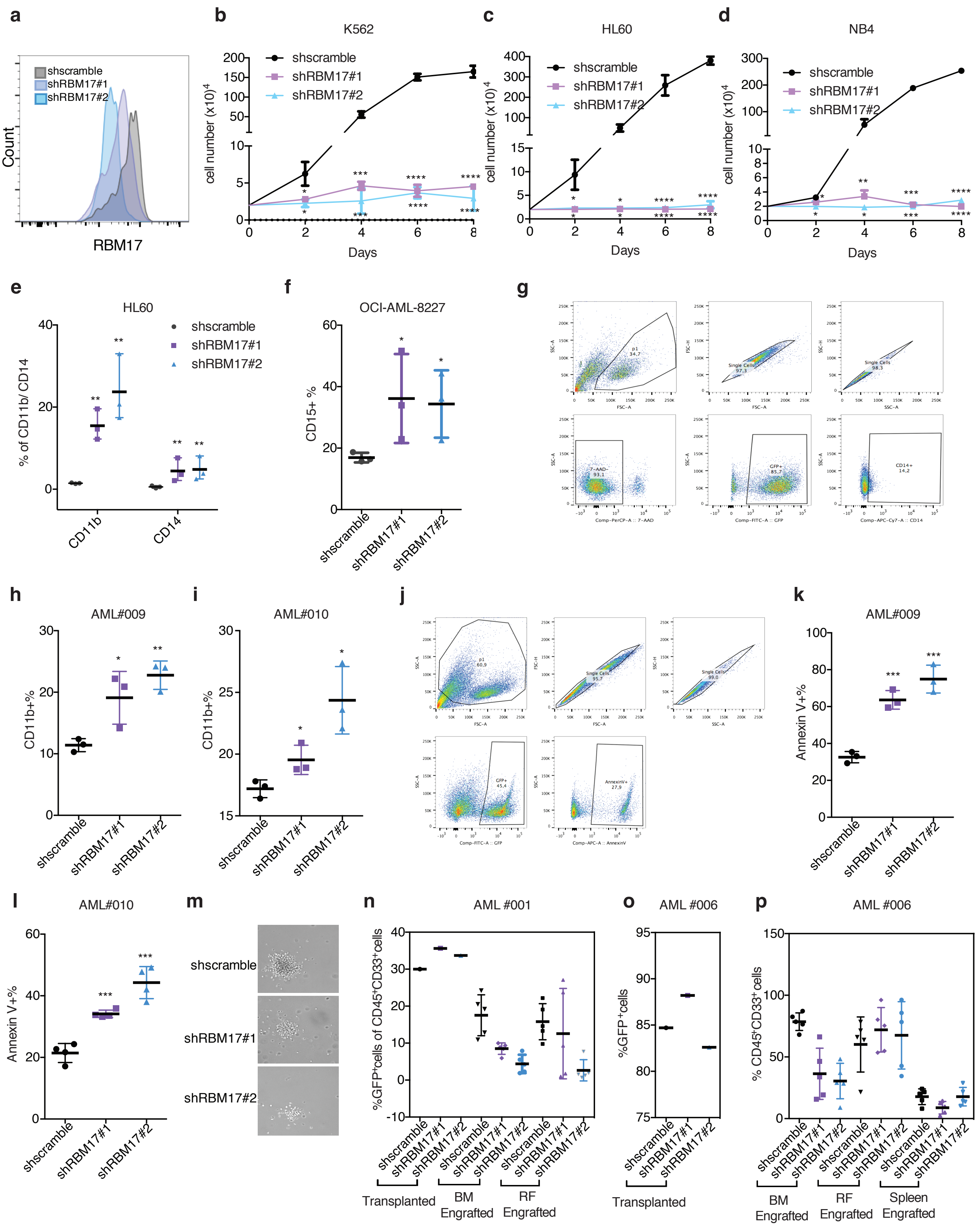
a
shscramble
shRBM17#1
shRBM17#2
Count
RBM17
b
K562
shscramble
shRBM17#1
shRBM17#2
cell number (x10)4
Days
c
HL60
d
NB4
e
HL60
% of CD11b/ CD14
CD11b
CD14
f
OCI-AML-8227
CD15+ %
g
7-AAD-
93.1
GFP+
85.7
CD14+
14.2
h
AML#009
CD11b+%
i
AML#010
j
GFP+
45.4
AnnexinV+
27.9
k
AML#009
Annexin V+%
l
AML#010
m
n
AML #001
%GFP+cells of CD45+CD33+cells
Transplanted
BM Engrafted
RF Engrafted
o
AML #006
%GFP+cells
p
AML #006
% CD45+CD33+ cells
Spleen Engrafted

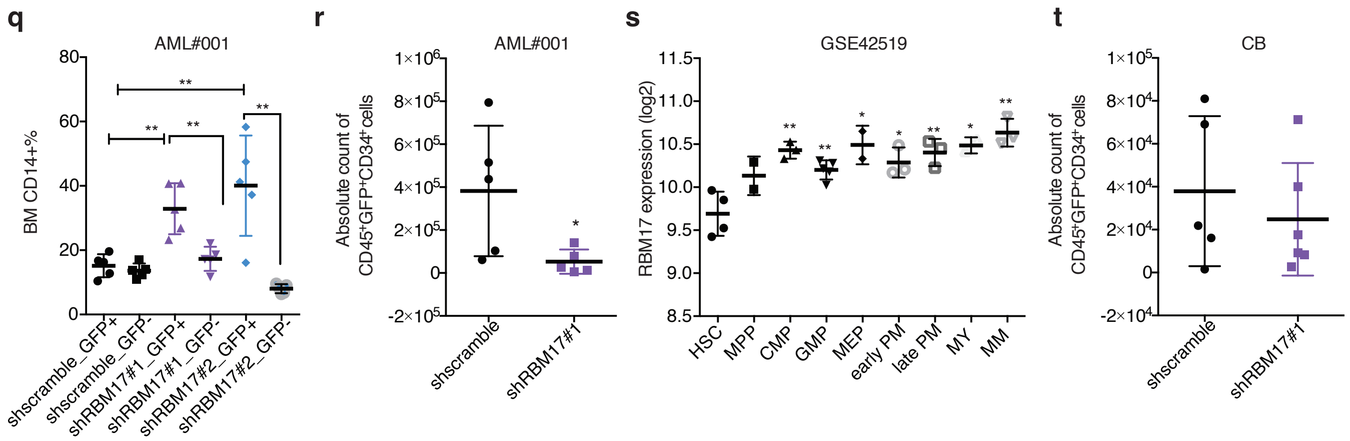

Supplementary Fig. 2: Related to Fig. 2. RBM17 supports AML cell proliferation and inhibits AML cell differentiation. (**a**) Flow cytometry validation of *RBM17* knockdown in CD34+ OCI-AML-8227 cells using shRBM17#1 and shRBM17#2. (**b-d**) Growth curves of transduced K562, HL60 and NB4 cells with *RBM17* knockdown and control lentivirus. *n*=3, mean ± SD. For each time point, knockdown groups are compared with the control shscramble group using two-tailed Student's *t* test. (**e-i**) Assessment of the effects of *RBM17* knockdown on myeloid differentiation of HL60 cells (**e**), OCI-AML-8227 cells (P(shscramble vs shRBM17#1)=0.0324, P(shscramble vs shRBM17#2)=0.0109) (**f**) and two primary AML samples (**h-i**) as compared to control shRNA. *n*=3, mean ± SD, two-tailed Student's *t* test. (**g**) One representative gating strategy applied to immunophenotyping assays, corresponding to figure 2**b**, 6**f**, supplementary figure 2**e-f**, 2**h-i** and supplementary figure 5d. (**j**) One representative gating strategy applied to Annexin V detection assay, corresponding to figure 2**c**, 6**g**, supplementary figure 2**k-l** and supplementary figure 5**e-g**. (**k-l**) Flow cytometry analysis of AnnexinV signal in primary AML cells on day 7 following knockdown of *RBM17*, mean ± SD, two-tailed Student's *t* test. AML#009: *n*=3, P(shscramble vs shRBM17#1)=0.0008, P(shscramble vs shRBM17#2)=0.0008. AML#010: *n*=4, P(shscramble vs shRBM17#1)=0.0003, P(shscramble vs shRBM17#2)=0.0003. (**m**) Example of colonies derived from primary AML cells transduced with a scramble (control) or RBM17-targeting shRNA (#1,2). Cells were sorted on the basis of GFP positivity 2 days after viral transduction and colonies were scored 14 d after plating in methylcellulose. (**n-p**) Raw data of engraftment experiment performed in AML sample #001 and sample #006, assessing the impact of *RBM17* knockdown on their engraftment potential. For sample #001, the y-axis (%GFP+ cells of CD45+ CD33+) indicates % of shRNA-positive cells in injected and engraftment human leukemia cells (**n**); For sample #006, shRNA infection rates are over 80% when injected (**o**) and the y-axis (%CD45+CD33+) indicates engraftment human leukemia cells in bone marrow (BM), right femur (RF) and spleen (**p**). *n*=5, mean ± SD, two-tailed Student's *t* test. (**q**) Immunophenotyping of myeloid differentiation in post-transplant grafts from sample#001 showing CD14 expression between GFP+ and GFP- cells in RBM17 knockdown groups and shscramble group. *n*=5, mean ± SD, two-tailed Student's *t* test. (**r**) Quantitative analysis of shRBM17-transduced AML cells at the endpoint showing the absolute cell number of CD34+ cells within positively engrafted mice compared to shscramble control mouse. *n*=5, mean ± SD, two-tailed Student's *t* test, P=0.0446. (**s**) *RBM17* mRNA expression in normal hematopoietic cells (GSE42519). mean ± SD, two-tailed Student's *t* test, P value was calculated as compared to HSC. (**t**) Quantitative analysis of shRBM17-transduced normal CD34+CD38- enriched HSCs at the endpoint showing the absolute cell number of CD34+ cells within positively engrafted mice compared to shscramble control mouse. *n*=5 (shscramble), *n=6* (shRBM17#1), mean ± SD, two-tailed Student's *t* test. *P<0.05, **P<0.01, ***P<0.001, ****P<0.0001.

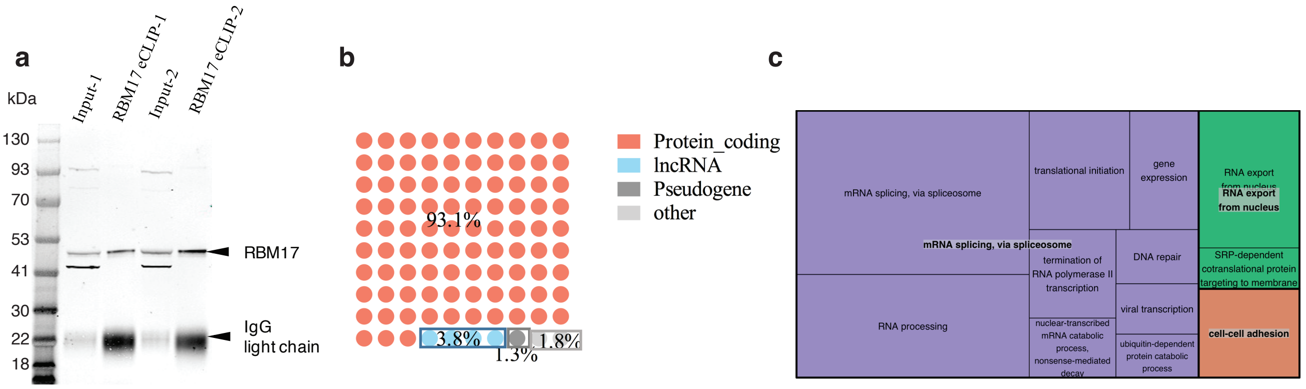

d

Validated 16 AS events impacted by RBM17 knockdown

Gene Symbol	AS event	Gene Symbol	AS event
MADD	Exon 16 exclusion	H2AFY	MXE
SRRM1	Exon 4 exclusion	EIF4A2	Intron 10 retention
MED24	Exon 6 exclusion	EZH2	Intron 9 retention
GSK3B	Exon 9 exclusion	MRPS18C	Exon 3 inclusion
MPZL1	Exon 5 exclusion	ZFYVE19	Exon 7 inclusion
ZDHHC3	Exon 5 exclusion	C4orf33	Exon 3 inclusion
PTPRC	Exon 4 exclusion	AP2B1	Exon 13 inclusion
RBM39	Exon 16 exclusion	USP33	Alt 3' SS

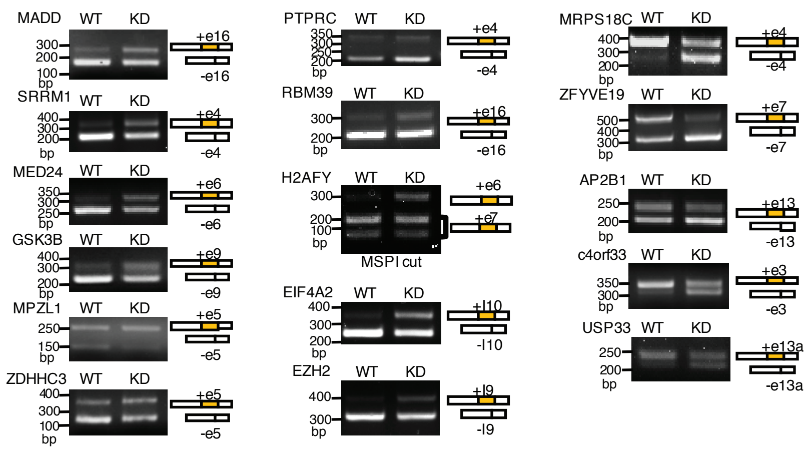

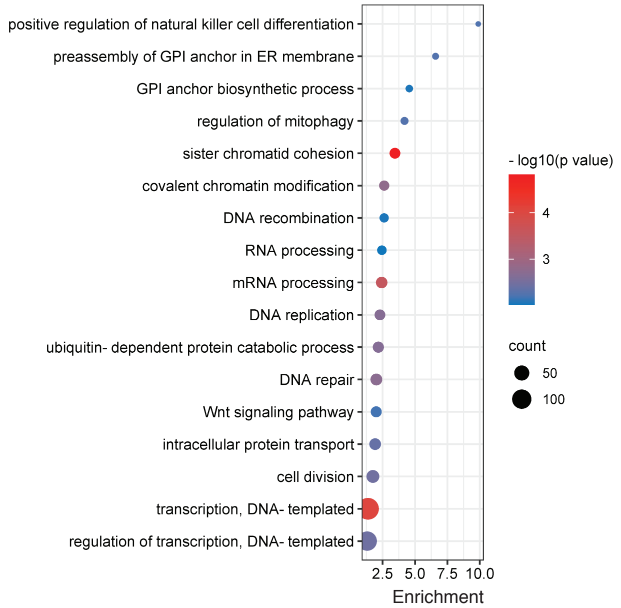

Supplementary Fig. 3: Related to Fig. 3. Identification of RBM17 RNA targets and downstream AS in AML. (a) Representative western blot showing results for the immunoblot analysis of the input and RBM17 samples, showed RBM17 is selectively immunoprecipitated. *n*=3 independent experiments. (**b**) Analysis of RBM17 binding gene type. (**c**) GO enrichment analysis of terms enriched in RNA targets bound by RBM17. (**d**) RT-PCR validated RBM17 regulated AS events in AML cells. (**e-f**) Representative RBM17-affected exon inclusion and exon skipping events validated by RT-PCR. The structure of each PCR product is indicated schematically on the right. Alternative exons affected by RBM17 are painted in orange. *n*=3 independent experiments. (**g**) Bubble plot showing GO pathway associations of genes with AS events downstream of RBM17. P value was calculated using modified Fisher's exact test. Source data are provided as a Source Data file.

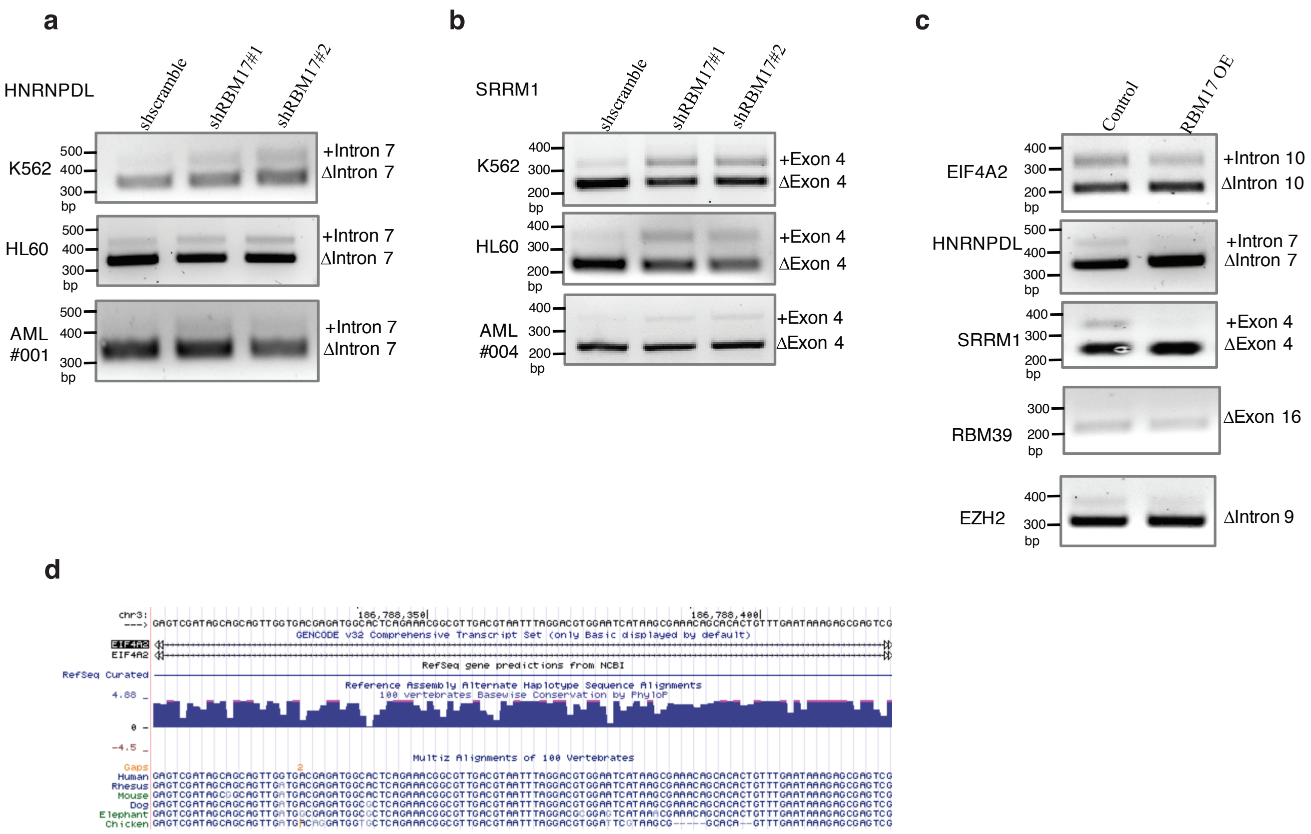

Supplementary Fig. 4: Related to Fig. 5. RBM17 knockdown leads to the production of NMD sensitive transcripts. (**a-b**) RT-PCR validation of *HNRNPDL* (**a**) and *SRRM1* (**b**) variants using RNA extracted from K562, HL60 and primary AML cells with and without *RBM17* knockdown. *n*=3 independent experiments. (**c**) RT-PCR validation of *EIF4A2*, *HNRNPDL*, *SRRM1*, *RBM39* and *EZH2* variants using RNA extracted from K562 cells with and without RBM17 overexpression. *n*=3 independent experiments. (**d**) The conservation track obtained from the UCSC Genome Browser shows high conservation of *EIF4A2* "cryptic exon" with PTC and flanking intronic sequences. Source data are provided as a Source Data file.

Supplementary Fig. 5

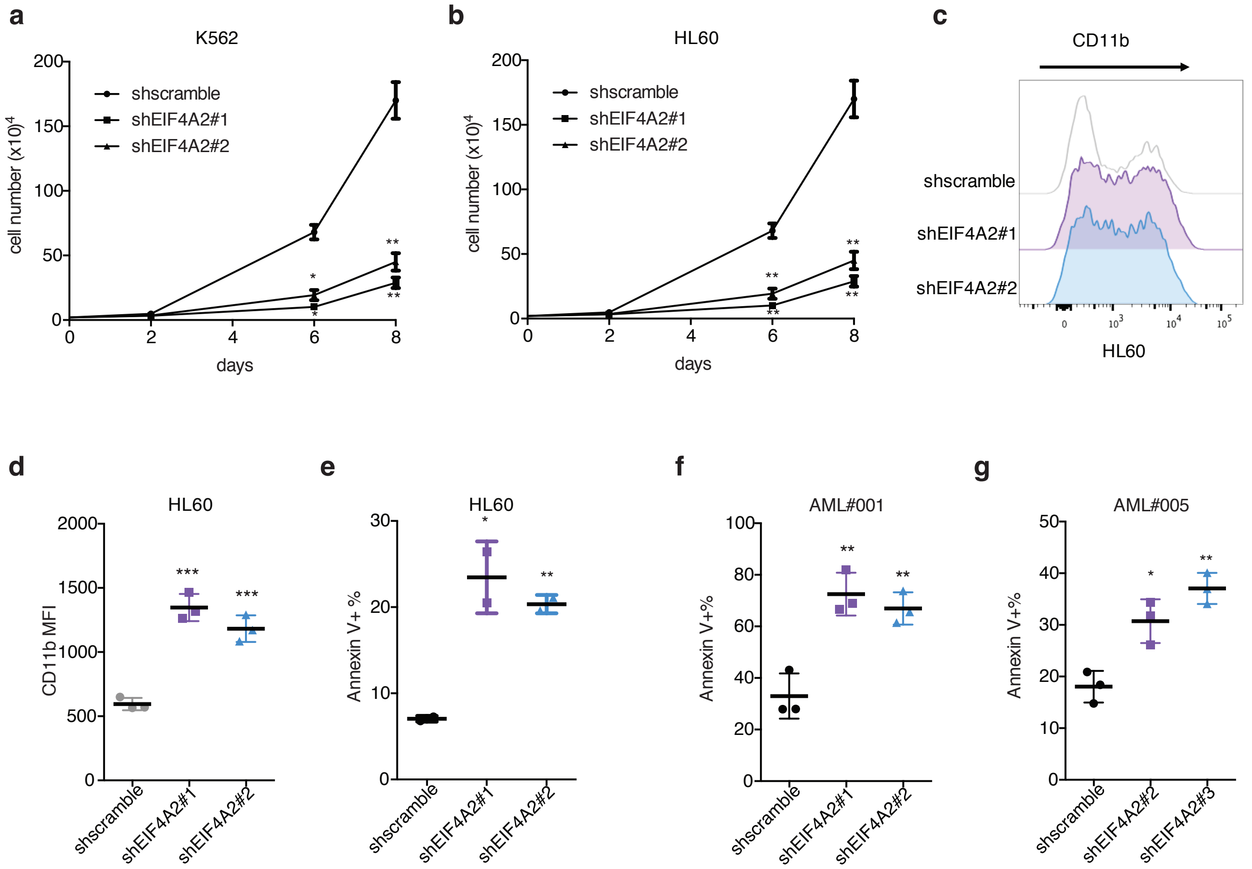

Supplementary Fig. 5: Related to Fig. 6. EIF4A2 is required for AML cell survival. (**a-b**) Assessment of the effects of *EIF4A2* knockdown on K562 (**a**) and HL60 (**b**) cell growth as compared to control shRNA cells. n=3, mean ± SD. For each time point, knockdown groups are compared with the control shscramble group using two-tailed Student's *t* test. (**c-e**) Flow cytometric evaluation of myeloid differentiation (P(shscramble vs shEIF4A2#1)=0.0004, P(shscramble vs shEIF4A2#2)=0.0009) (**c-d**) and apoptosis (P(shscramble vs shEIF4A2#1)=0.031, P(shscramble vs shEIF4A2#2)=0.0035) (**e**) following *EIF4A2* knockdown in HL60 cells. n=3, mean ± SD, two-tailed Student's *t* test. (**f-g**) Flow cytometric evaluation of apoptosis following *EIF4A2* knockdown in primary AML cells. n=3, mean ± SD, two-tailed Student's *t* test. *P<0.05, **P<0.01, ***P<0.001.

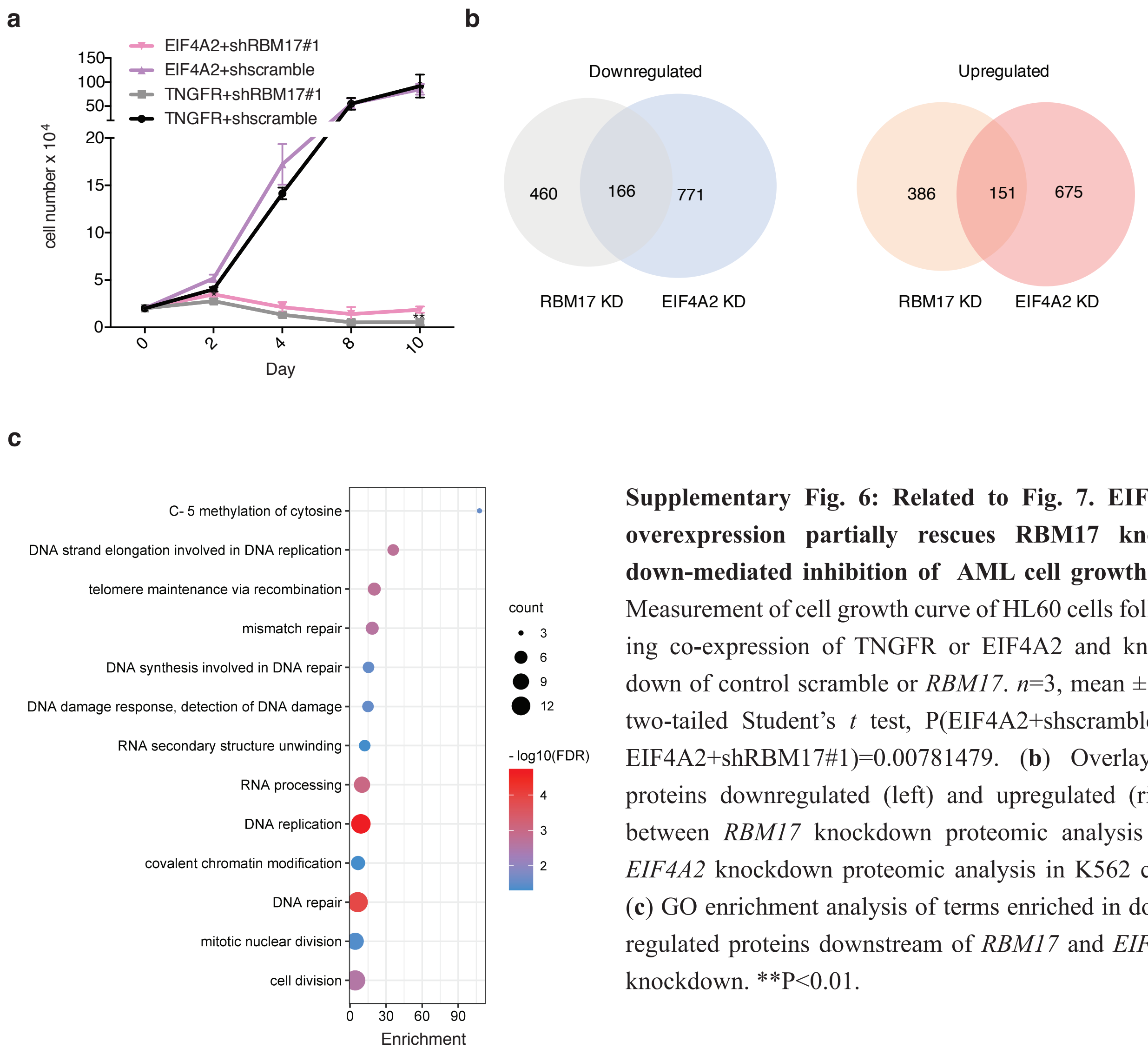

Supplementary Fig. 6: Related to Fig. 7. EIF4A2 overexpression partially rescues RBM17 knock-down-mediated inhibition of AML cell growth. (**a**) Measurement of cell growth curve of HL60 cells following co-expression of TNGFR or EIF4A2 and knock-down of control scramble or *RBM17*. *n*=3, mean ± SD, two-tailed Student's *t* test, P(EIF4A2+shscramble vs EIF4A2+shRBM17#1)=0.00781479. (**b**) Overlay of proteins downregulated (left) and upregulated (right) between *RBM17* knockdown proteomic analysis and *EIF4A2* knockdown proteomic analysis in K562 cells. (**c**) GO enrichment analysis of terms enriched in down-regulated proteins downstream of *RBM17* and *EIF4A2* knockdown. **P<0.01.

Supplementary Table 1:
Clinical characteristics of primary AML patient samples

sample#	Diagnosis	Cytogenetics	FAB subtype	Additional molecular markers
001	De novo	46,XY,ider(7)(q10) del(7)(q21)[20]	M1	not done
002	De novo	NA	M5	NA
003	MDS, refractory cytopenia with multilineage dysplasia	47,XY,+8[2]/46, XY[20]	NA	not done
004	AML, sub-type unclassified	47,XX,+8[14]	NA	FLT3-ITD
005	De novo	46, XY[20]	M1	NA
006	De novo	46, XY[21]	M1	FLT3-ITD
007	De novo	46, XY[20]	M1	not done
008	De novo	47, XY[20]	M0	not done
009	De novo	46, XY[20]	M4/M5-myelomonocytic with MDS related changes	NPM1 and high FLT3-ITD
010	Relapse	46, XX[20]	Monocytic differentiation (favor M4)	NPM1, FLT3-ITD and FLt3-TKD

Supplementary Table 2:
GSEA of genes co-regulated with RBM17 in human AML

Go term	SIZE	NES	NOM p-val	FDR q-val
GO_RIBONUCLEOPROTEIN_COMPLEX_BIOGENESIS	431	2.0012367	0	0.021558888
GO_RIBOSOMAL_LARGE_SUBUNIT_BIOGENESIS	72	2.0189736	0	0.023335185
GO_DESMOSOME	24	2.0468068	0	0.024645623
GO_GOLGI_CIS_CISTERNA	24	1.9186343	0	0.027782617
GO_TRNA_METHYLATION	40	1.920206	0	0.029331803
GO_RNA_METHYLATION	82	1.9078909	0	0.030454688
GO_MATURATION_OF_LSU_RRNA	27	1.9297539	0	0.030612184
GO_TRNA_METHYLTRANSFERASE_ACTIVITY	34	1.923328	0	0.030679988
GO_RIBOSOME_BIOGENESIS	300	1.931395	0	0.034042753
GO_AXON_INITIAL_SEGMENT	16	1.9620454	0	0.035222724
GO_PRERIBOSOME	75	1.8922114	0	0.038252123
GO_RNA_METHYLTRANSFERASE_ACTIVITY	67	1.9330649	0	0.038755246
GO_VOLTAGE_GATED_SODIUM_CHANNEL_COMPLEX	17	1.9355748	0	0.044540383
GO_SPLICEOSOMAL_COMPLEX_ASSEMBLY	59	1.8475881	0	0.04826988

Supplementary Table 3: RT-PCR primer sequences

Target genes	Forward primer (5'-3')	Reverse primer (5'-3')
MADD	AACTCCACCGTCTCCAACAC	CACTAACGCCCTCCTGTTTC
SRRM1	ACGGAAATCCTTGGGTTTGA	AGGAAAGCAGAAGGGATTCCC
MED24	CACGGCAAAGCAGAGGAATG	AATGCTCGATGGCAGTCCAA
GSK3B	CCTGGGAACTCCAACAAGGG	AAGAGTGCAGGTGTGTCTCG
EIF4A2	GGTCAGGGTCAAGTCGTGTT	TGGGCATCTCCTCCACTGTA
EZH2	TCATGCAACACCCAACACTT	AGAGGAGCTCGAAGTTTCATCT
MPZL1	AGCATGATTCTGGCTGTCCT	GGATATCCGCATACACCACA
ZDHHC3	ACTGTCCCTGGGTCAACAAC	GTGGAGAGAAGGAGCTGCAC
PTPRC	GTATTTGTGACAGGGCAAAGC	AGAGTGGTTGTTTCAGAGGCA
H2AFY	GACGGCTTCACAGTCCTCTC	TCCAAGGGCCCGTTCTTTTT
RBM39	CAAGTTGAGCAGTGCCAACG	ATCTCGACTTCTTGAGCGGC
MRPS18C	TTGCTGTTTGCGGTGGTCTA	TGTAACTGGCATAAACCCCAT
ZFYVE19	CAGAGATAGAGGCACGGCTG	GCAGGACTCTTTGGATGGCT
C4orf33	TGCTCCATTTTTCAGGGATCCT	TGCTCCATTTTTCAGGGATCCT
USP33	TGTCTGACTTGTGACAGGGTG	GCAAGGTTACTACTGGACCCC
AP2B1	AATGTGCCACAGGTGTCCTC	ATATCCACCAGGTGCCATGC
HNRNPDL	ATCTCGAGGGGGTGGCAAT	AGTACCTGACGCAGAAAAGCA

Supplementary Table 4: Oligonucleotides used for cloning

Gene name	Forward primer (5'-3')	Reverse primer (5'-3')
shEIF4 A2#1	CCGGAGGCGATCACAACGTGCATTGCTCGAGCAATG CACGTTGTGATCGCCTTTTTTG	AATTCAAAAAAGGCGATCACAACGTGCATTGCTCGA GCAATGCACGTTGTGATCGCCT
shEIF4 A2#2	CCGGGCCAGAGACTTCACAGTTTCTCTCGAGAGAAA CTGTGAAGTCTCTGGCTTTTTG	AATTCAAAAAGCCAGAGACTTCACAGTTTCTCTCGAG AGAAACTGTGAAGTCTCTGGC
shUPF 1#1	CCGGTTACCTTGGTGACGAGTTTAACTCGAGTTAAAC TCGTCACCAAGGTAATTTTTG	AATTCAAAAATTACCTTGGTGACGAGTTTAACTCGAG TTAAACTCGTCACCAAGGTAA
shUPF 1#2	CCGGAGATATGCCTGCGGTACAAAGCTCGAGCTTTG TACCGCAGGCATATCTTTTTTG	AATTCAAAAAAGATATGCCTGCGGTACAAAGCTCGA GCTTTGTACCGCAGGCATATCT
shLuci	CCGGCGCTGAGTACTTCGAAATGTCCTCGAGGACAT TTCGAAGTACTCAGCGTTTTTg	AATTCAAAAACGCTGAGTACTTCGAAATGTCCTCGA GGACATTTCGAAGTACTCAGCG
EGFP	CGCGGATCCATGGTGAGCAAGGGCGAGGA	CGGGGTACCTTACTTGTACAGCTCGTCCA
EIF4A2	CGCGGATCCaccATGTCTGGTGGCTCCGCG	CGCGGATCCTTAAATAAGGTCAGCCACATTC

Supplementary Table 5: qPCR primer sequences

Target gene	Forward primer (5'-3')	Reverse primer (5'-3')
UPF1	AATTTGGTTAAGAGACATGCGG	TCAGGGACCTTGATGACGTG
18srRNA	GTAACCCGTTGAACCCCATT	CCATCCAATCGGTAGTAGCG
EIF4A2_Intron10 included isoform	TAGCAGCAGTTGGTGACGAG	CGACTCGCTCTTTATTCAAACA
EIF4A2_Intron10 skipped isoform	TATTCACAGAATTGGCAGAGG	TGGGCATCTCCTCCACTGTA
RBM17	TGAGCGAGAGAGGAGGAAAA	CCATGTTAGCGAGGAAGGAG
