# Supplementary material for: The splicing factor RBM17 drives leukemic stem cell maintenance by evading nonsense-mediated decay of pro-leukemic factors
Source: Nat Commun. 2022 Jul 4;13:3833. doi: 10.1038/s41467-022-31155-0 (PMC9250932; doi:10.1038/s41467-022-31155-0)
Supplement: Supplementary file 2 — Description of Additional Supplementary Files [file 41467_2022_31155_MOESM2_ESM.pdf]

Description of Additional Supplementary Files

File Name: Supplementary Data 1
Description: AML prognostic P vlaues, LSC+/LSC- P values and average expressions in LSC+ versus LSC- of 203 mRNA splicing factors. Related to Figure 1.

File Name: Supplementary Data 2
Description: 866 significantly enriched reproducible binding peaks for RBM17 in the genome identified from RBM17 eCLIP-seq in K562 cells. Related to Figure 3.

File Name: Supplementary Data 3
Description: Differentially spliced events in K562 cells treated with shRBM17 versus control shscramble. Related to Figure 3.

File Name: Supplementary Data 4
Description: Splicing events directly bound by RBM17. Related to Figure 3.

File Name: Supplementary Data 5
Description: Coding potential and protein domain changes prediction of splicing events affected by RBM17 knockdown in K562 cells. Related to Figure 4.

File Name: Supplementary Data 6
Description: Differentially expressed proteins in K562 cells treated with shRBM17 versus control shscramble. Related to Figure 4.

File Name: Supplementary Data 7
Description: Proteins included in the EIF4A2_KD_DN and EIF4A2_KD_UP gene set. Related to Figure 7.

File Name: Supplementary Data 8
Description: Protein expression changes of 22 translation related factors in K562 cells with and without EIF4A2 knockdown or RBM17 knockdown. Related to Figure 7.
