# Supplementary material for: The splicing factor RBM17 drives leukemic stem cell maintenance by evading nonsense-mediated decay of pro-leukemic factors
Source: Nat Commun. 2022 Jul 4;13:3833. doi: 10.1038/s41467-022-31155-0 (PMC9250932; doi:10.1038/s41467-022-31155-0)
Supplement: Supplementary file 11 — Reporting Summary [file 41467_2022_31155_MOESM11_ESM.pdf]

nature portfolio

Corresponding author(s): Dr. Yu Lu, Dr.Kristin Hope

Last updated by author(s): Apr 20, 2022

Reporting Summary

Nature Portfolio wishes to improve the reproducibility of the work that we publish. This form provides structure for consistency and transparency in reporting. For further information on Nature Portfolio policies, see our Editorial Policies and the Editorial Policy Checklist.

Statistics

For all statistical analyses, confirm that the following items are present in the figure legend, table legend, main text, or Methods section.

n/a	Confirmed	
☐	☒	The exact sample size (*n*) for each experimental group/condition, given as a discrete number and unit of measurement
☐	☒	A statement on whether measurements were taken from distinct samples or whether the same sample was measured repeatedly
☐	☒	The statistical test(s) used AND whether they are one- or two-sided<br>*Only common tests should be described solely by name; describe more complex techniques in the Methods section.*
☐	☒	A description of all covariates tested
☐	☒	A description of any assumptions or corrections, such as tests of normality and adjustment for multiple comparisons
☐	☒	A full description of the statistical parameters including central tendency (e.g. means) or other basic estimates (e.g. regression coefficient) AND variation (e.g. standard deviation) or associated estimates of uncertainty (e.g. confidence intervals)
☐	☒	For null hypothesis testing, the test statistic (e.g. F, t, r) with confidence intervals, effect sizes, degrees of freedom and P value noted<br>*Give P values as exact values whenever suitable.*
☒	☐	For Bayesian analysis, information on the choice of priors and Markov chain Monte Carlo settings
☐	☒	For hierarchical and complex designs, identification of the appropriate level for tests and full reporting of outcomes
☐	☒	Estimates of effect sizes (e.g. Cohen's *d*, Pearson's *r*), indicating how they were calculated

Our web collection on statistics for biologists contains articles on many of the points above.

Software and code

Policy information about availability of computer code

Data collection	GraphPad Software (v6.0);<br>FlowJo Software (v7.6.5);<br>Cytoscape Software (v3.8.2);<br>GSEA Software (v4.1.0);<br>IGV Software (v2.12.2);<br>Image Lab (v6.0.1);<br>FACSDiva (v9.0.1);
Data analysis	For RNA-seq data, quality control and basic pre-processing were done using FastQC (v0.11.5), the FastxToolkit (v0.0.13) and trimmomatic v0.38. Reads were aligned to the human genome (UCSC hg38) using the transcriptome aligner STAR v 2.7.2b. Alternative splicing analyses were done using rMATS (v 4.0.1). Standard settings were used. Predictions for the functional effects of alternative splicing were made using Python scripts, as described previously (Anande et al, Clinical Cancer Research (2020): 26 (14), 3597-3607). The Bioconductor/R packages maser and drawProteins were used for visualizations.<br>For RBM17 eCLIP-seq data analysis, Fastq files were run through eCLIP-v0.4.0 pipeline as described previously (Van Nostrand et al., 2017).<br>LC-MS data generated was analyzed against a UniProt human protein database (42,173 entries) for protein identification and quantification by Thermo Proteome Discoverer (v 2.2.0). Identified proteins have at least one unique peptide. The FDR was calculated from the output P values using Benjamini-Hochberg method. The fold change (FC) of normalized protein expression intensities (FC<0.9 or FC>1.1) and FDR<0.1 was used to identify proteins that are differentially abundant and used for downstream integrative analysis.

For manuscripts utilizing custom algorithms or software that are central to the research but not yet described in published literature, software must be made available to editors and reviewers. We strongly encourage code deposition in a community repository (e.g. GitHub). See the Nature Portfolio guidelines for submitting code & software for further information.

March 2021

Data

Policy information about availability of data

All manuscripts must include a data availability statement. This statement should provide the following information, where applicable:

- Accession codes, unique identifiers, or web links for publicly available datasets
- A description of any restrictions on data availability
- For clinical datasets or third party data, please ensure that the statement adheres to our policy

eCLIP-seq data have been deposited in NCBI's GEO and are accessible through GEO Series accession number GSE180955. The mass spectrometry proteomics raw data have been deposited in the ProteomeXchange Consortium via Proteomics Identification (PRIDE). The accession number of the proteomics data reported in this paper is PRIDE: PXD026780. Gene expression data of 138 xenotransplant defined LSC-enriched and 89 non-LSC subsets from 78 AML were obtained from GEO (GSE76008). Gene expression data on sorted LT-HSC (most primitive hematopoietic cells) from AML patients and healthy controls were obtained from GEO (GSE35008). Protein expression of xenotransplant validated LSC-enriched and non-LSC fractions from 6 AML samples were obtained from PRIDE (Project PXD008307). Gene set enrichment analysis (GSEA) was performed by compassion of the "RBM17 high" and "RBM17 low" gene expression profile with the published LSC gene signature (GSE76008). RNA-seq data of shRBM17 or Control transduced K562 cells were downloaded from GEO (GSE88633) and GEO (GSE88047). Gene expression data of normal human hematopoietic cells were obtained from GEO (GSE42519). Leucegene AML dataset were obtained from GEO (GSE67040), BeatAML dataset can be accessed through the link https://www.cbioportal.org/, LAML-TCGA dataset can be accessed through the link https://portal.gdc.cancer.gov/projects/TCGA-LAML.

Field-specific reporting

Please select the one below that is the best fit for your research. If you are not sure, read the appropriate sections before making your selection.

☒ Life sciences ☐ Behavioural & social sciences ☐ Ecological, evolutionary & environmental sciences

For a reference copy of the document with all sections, see nature.com/documents/nr-reporting-summary-flat.pdf

Life sciences study design

All studies must disclose on these points even when the disclosure is negative.

Sample size	The number of samples for each assay was indicated in each figure legend. For in vitro cellular and biochemical assays, no statistical methods were used to predetermine sample sizes, however the sample sizes were chosen based on our experience with these same assays yielding statistically significant differences between experimental positive and negative controls and on similar sample sizes used in published literature. Results are representatives of at least three independent experiments. For in vivo AML/CB transplant assays, 5~6 mice were chosen for each condition, this sample size was determined by using power calculation for a t-test difference between two independent means based on a normally distributed population with equal variance (a significance level of 0.05, an effect size of 2 and a power of 0.80), and also based on similar studies of our own and in the field (ie. Ly et al., Cancer Research, 2019; Jones et al., Cell Stem Cell, 2020).
Data exclusions	The Grubb's outlier analysis was done for Figure 6C and the data point at y=3.716161 was found to be an outlier value. As such we have removed it from the Figure 6C.
Replication	A minimum of independent triplicates were carried out for each experiment as described in the legends for each figure.
Randomization	Animals were randomly divided into experimental groups. All other samples collected were used for this study without any discrimination.
Blinding	Blinding was not used in this study. Complex and time intensive genetic manipulation (Knockdown, overexpression) was performed by the same individual skilled in specific functional evaluations. The absence of multiple individuals with completely overlapping skill sets therefore meant that authors were aware of experimental groups.

Reporting for specific materials, systems and methods

We require information from authors about some types of materials, experimental systems and methods used in many studies. Here, indicate whether each material, system or method listed is relevant to your study. If you are not sure if a list item applies to your research, read the appropriate section before selecting a response.

Materials & experimental systems

n/a	Involved in the study	
☐	☒	Antibodies
☐	☒	Eukaryotic cell lines
☒	☐	Palaeontology and archaeology
☐	☒	Animals and other organisms
☐	☒	Human research participants
☒	☐	Clinical data
☒	☐	Dual use research of concern

Methods

n/a	Involved in the study	
☒	☐	ChIP-seq
☐	☒	Flow cytometry
☒	☐	MRI-based neuroimaging

Antibodies

Antibodies used

Flow cytometry: anti-CD34 APC (clone 581, cat#555824, BD Biosciences); PE-CD11b (clone ICRF44, cat#557321, BD); BV605-CD11b (clone ICRF44, cat#562721, BD); FITC- CD14 (clone HI98, cat#551376, BD); APC-H7-CD14 (clone M5E2, cat#561384, BD); APC-CD15 (clone HI98, cat#551376, BD); BV421-CD45 (clone HI30, at#563879, BD); PE-CD33 (clone P67.6, cat#347787, BD); anti-RBM17 (cat#ab204333, Abcam);
Western blot: anti-RBM17 (A302-498A, Bethyl); anti-EIF4A2 (NBP2-24612SS, Novus Biologicals); anti-GAPDH (2118S, New England BioLabs), IRDye 680RD Goat anti-Rabbit IgG (LIC-925-68071, Mandel Scientific), IRDye 800CW Goat anti-Rabbit IgG (LIC-925-32211, Mandel Scientific); IRDye 800CW Donkey anti-Mouse IgG (H+L) antibody (LIC-92632212, Mandel Scientific), Mouse Anti-rabbit IgG (Light-Chain Specific) (D4W3E) mAb (cat#45262S, New England BioLabs);
eCLIP/IP: anti-RBM17 (A302- 497A and A302-498A, Bethyl);

Validation

Antibodies were chosen based on the validation statements for the species (human) and application (WB, IP or FACS) on the manufacturer's website.
anti-EIF4A2 (NBP2-24612SS, Novus Biologicals) and anti-GAPDH (2118S, New England BioLabs) antibodies have been validated by the manufacturer. Anti-RBM17 (A302-498A, Bethyl) antibody has been validated by Bethyl by immunoblotting on Hela cell lysate and by IP on 1mg Hela cell lysate. This antibody has also been used for RBM17 eclip-seq in HepG2 cell line in a previous study(Van Nostrand et al., 2020). Anti-RBM17 (A302-497A, Bethyl) antibody has been validated by Bethyl for IP experiment on 1mg HEK293T cell lysate. Equal amount of anti-RBM17 (A302- 497A and A302-498A) antibody mixture has been validated in the previous study (De Maio et al., 2018) on RBM17 IP-MS performed on C57BL/6J male mice cerebella.

Eukaryotic cell lines

Policy information about cell lines

Cell line source(s)

HEK293FT (CRL-1573), K562 (CCL-243) and HL60 (CCL-240) cell lines were obtained from ATCC. The NB4 cell line (ACC 207) was obtained from DSMZ. The LentiX-293T cell line (632180) was obtained from Clontech/Takara. OCI-AML8227 patient-derived model was a gift from Dr. Eric Lechman. OCI-AML8227 cell was derived from a relapse AML patient sample that is organized as a functional hierarchy (Lechman et al., 2016, Cancer Cell 29, 214-228).

Authentication

Identities of K562, HL60 cell lines were confirmed with matched STR profiles using CellCheck 9TM Plus (IDEXX bioanalytics). 8227 cells were checked by cell surface phenotype. NB4, HEK293FT and LentiX-293T cell lines were not authenticated.

Mycoplasma contamination

All cell lines were tested negative for mycoplasma contamination.

Commonly misidentified lines
(See ICLAC register)

No commonly misidentified cell lines were used.

Animals and other organisms

Policy information about studies involving animals; ARRIVE guidelines recommended for reporting animal research

Laboratory animals

NOD-scid-IL2Rgc / (NSG; Jackson Laboratory) mice (male and female), age week 6-7 weeks old were used for in vivo primary AML transplants.The housing lights are on a 12 hours on/ 12 hours off cycle (6 am on and 6 pm off everyday), the housing temperature is between 19-21? and the humidity is 40~60%.

Wild animals

No wild animals were used in this study.

Field-collected samples

No field-collected samples were used in this study.

Ethics oversight

All procedures were approved by the Animal Research Ethics Board at McMaster University, HiREB (Hamilton Integrated Research Ethics Board), the Canadian Counsel on Aminal Care and the University Health Network Animal Care Committee.

Note that full information on the approval of the study protocol must also be provided in the manuscript.

Human research participants

Policy information about studies involving human research participants

Population characteristics	Relevant clinical information of human research participants was included in supplementary table 2.
Recruitment	Participants were recruited in an unbiased manner.
Ethics oversight	All patient samples were obtained with written informed consent and with the approval of the local human subject research ethics board at the University Health Network and McMaster University in accordance with Canadian Tri-Council Policy Statement on the Ethical Conduct for Research Involving Humans (TCPS).

Note that full information on the approval of the study protocol must also be provided in the manuscript.

Flow Cytometry

Plots

Confirm that:

☒ The axis labels state the marker and fluorochrome used (e.g. CD4-FITC).

☒ The axis scales are clearly visible. Include numbers along axes only for bottom left plot of group (a 'group' is an analysis of identical markers).

☒ All plots are contour plots with outliers or pseudocolor plots.

☒ A numerical value for number of cells or percentage (with statistics) is provided.

Methodology

Sample preparation

For RBM17 intracellular flow, primary AML cells and 8227 cells were initially stained with LIVE/DEAD Fixable Green (L34969, Thermo Fisher) and anti-CD34 APC (555824, BD Biosciences) antibody and then fixed with the Cytofix/Cytoperm kit (BD Biosciences) according to the manufacturer's instructions. Fixed and permeabilized cells were immunostained with anti-RBM17 rabbit antibody (ab204333, Abcam) and detected by Alexa-Fluor 405 goat anti-rabbit IgG(H+L) secondary antibody (Thermo Fisher) through FCAS. For RBM17 knockdown efficiency test, sorted GFP positive cells were initially stained with LIVE/DEAD Fixable Near-IR (L34975, Thermo Fisher) for 30 minutes and then fixed with the Cytofix/Cytoperm kit. Fixed and permeabilized cells were immunostained with anti-RBM17 rabbit antibody and detected the same way as described above.

To measure apoptosis, cells were washed with PBS and incubated with 5 µl of Annexin V- FITC (APOAF, sigma) or anti-V450-Annexin V (560506, BD) and 1.25 µl of PI or 7-AAD in 1X Annexin V binding buffer in a reaction volume of 100ul for 15 minutes at room temperature, in the dark. Cells were then topped up with 200ul of 1X Annexin V binding buffer and analyzed on the LSRII (BD Biosciences). To monitor cellular differentiation status, cells were washed with PBS and then stained with the following antibodies: PE-CD11b (557321, BD), FITC- CD14 (551376, BD) or APC-H7-CD14 (561384, BD) and APC-CD15 (551376, BD) for 20 minutes at dark. Then cells were washed with PEF twice and resuspended in 200ul of 7-AAD (1:75) PEF.

To analyze AML transduced mice bone marrow cells at the end point of the experiment, bone marrow from tibias, femurs and pelvis was harvested, crushed with mortar and pestle, filtered and red blood cell lysed using ammonium chloride buffer. Human AML engraftment was analyzed by blocking reconstituted mouse bone marrow with mouse 1:50 rat anti-human Fc block (BD Biosciences) and 1:10 human IgG (Sigma-Aldrich), followed by staining with fluorochrome-conjugated antibodies against human CD45-BV-421, CD33-PE, CD34-APC, CD14-APC-H7, CD11b-BV605 (BD Biosciences) for graft analysis.

Instrument	MACSQuant Flow Cytometer, BD LSRII, BD LSR Fortessa, BD FACS Aria II Cell Sorter, BD FACS Aria III and Beckman Mo-Flo Astrios.
Software	FACS Diva (v9.0.1) for data collection. Flowjo (v10.2).
Cell population abundance	The abundance of post-sort fractions were higher than 95%. The purity was determined by analyzing 7-AAD-GFP+% cell population in post-sorted samples.

Gating strategy

For Annexin V detection assay, cell debris were excluded using FSC and SSC, then doublets were excluded using FSC-A and FSC-H, transduced GFP-positive cells were selected and Annexin V-positive cells were further determined.
All other samples were initially gated using the FSC/SSC to identify events corresponding to cells and remove debris, then singlets were selected using FSC-A and FSC-H and live cells were further enriched by gating on LIVE/DEAD Fixable Green/ NearIR –negative or 7-AAD-negative cells.
1) For RBM17 intracellular flow, within live cell population, CD34-postive and CD34-negative cells were gated based on unstained control. Then RBM17-positive and RBM17-negative cells were furthered gated based on unstained cells + Alexa-Fluor 405 goat anti-rabbit IgG(H+L) secondary antibody control within CD34-positive and CD34-negative cells.
2) For in vitro immunophenotyping assays, transduced GFP-positive cells were selected within live cell population and then CD14, CD15, CD11b positivity were determined. Gating strategy of in vivo immunophenotyping analyses of grafts from primary AML transplanted mice were described in the Supplementary method section.

☒ Tick this box to confirm that a figure exemplifying the gating strategy is provided in the Supplementary Information.
